# Oral misoprostol, low dose vaginal misoprostol, and vaginal dinoprostone for labor induction: Randomized controlled trial

**DOI:** 10.1371/journal.pone.0227245

**Published:** 2020-01-10

**Authors:** David C. Young, Tina Delaney, B. Anthony Armson, Cora Fanning

**Affiliations:** 1 Department of Obstetrics & Gynaecology, Dalhousie University, Halifax, Nova Scotia, Canada; 2 Department of Obstetrics & Gynaecology, IWK Health Centre, Halifax, Nova Scotia, Canada; 3 Department of Obstetrics & Gynaecology, Memorial University of Newfoundland, St. John’s, Newfoundland and Labrador, Canada; Centre Hospitalier Departementai Vendee, FRANCE

## Abstract

**Objective:**

To compare effectiveness and safety of oral misoprostol (50 μg every four hours as needed), low dose vaginal misoprostol (25 to 50 μg every six hours as needed), and our established dinoprostone vaginal gel (one to two mg every six hours as needed) induction.

**Materials and methods:**

Consenting women with a live term single cephalic fetus for indicated labor induction were randomized (3N = 511). Prior uterine surgery or non-reassuring fetal surveillance were exclusions. Concealed computer generated randomization was stratified and blocked. Newborns were assessed by a team unaware of group assignment. The primary outcome was time from induction at randomization to vaginal birth for initial parametric analysis. Sample size was based on mean difference of 240 minutes with α_2_ = 0.05 and power 95%. Non-parametric analysis was also pre-specified ranking cesareans as longest vaginal births.

**Results:**

Enrollment was from April 1999 to December 2000. Demographics were similar across groups. Analysis was by intent to treat, with no loss to follow up. Mean time (±SD) to vaginal birth was 1356 (±1033) minutes for oral misoprostol, 1530 (±3249) minutes for vaginal misoprostol, and 1208 (±613) minutes for vaginal dinoprostone (P = 0.46, ANOVA). Median times to vaginal birth were 1571, 1339, and 1451 minutes respectively (P = 0.46, Kruskal-Wallis). Vaginal births occurred within 24 hours in 44.9, 53.5 and 47.7% respectively (P = 0.27, χ^2^). There were no significant differences in Kaplan Meier survival analyses, cesareans, adverse effects, or maternal satisfaction. The newborn who met birth asphyxia criteria received vaginal misoprostol, as did. all three other newborns with cord artery pH<7.0 (P = 0.04, Fisher Exact).

**Conclusion:**

There was no significant difference in effectiveness of the three groups. Profound newborn acidemia, though infrequent, occurred only with low dose vaginal misoprostol.

## Introduction

Induction of labor is its intentional initiation before spontaneous onset, with the aim of vaginal birth which is safe for mother and newborn. Established indications for induction include post-term pregnancy, pre-labor membrane rupture (PROM), and maternal hypertension. A frequent obstetric intervention, current rates [1–3] exceed 20% of births. Induction rates are likely to rise following the recent compelling randomized controlled trial (RCT) evidence [4] where lower cesarean rates (18.6% versus 22.2%, relative risk[RR] 0.84, 95% confidence interval[CI] 0.76–0.93; P<0.001) were achieved with elective induction of low risk nulliparas at 39 weeks gestation, versus expectant management. Notably though not reaching pre-specified levels for statistical significance, there was also a 20% reduction in the primary composite outcome of perinatal mortality and severe perinatal morbidity (RR 0.80, 95% CI 0.64–1.00; P = 0.049). In addition, induction subjects had significantly lower rates of gestational hypertension and preeclampsia (9.1% versus 14.1%, RR 0.64, 95% CI 0.56–0.74; P< 0.001), as well as need for neonatal respiratory support within 72 hours of birth (3.0% versus 4.2%, RR 0.71, 95% CI 0.55–0.93). A specific induction method was not mandated, however cervical ripening by a protocol at the attending physician’s discretion was encouraged prior to oxytocin stimulation [4].

Dilute intravenous (IV) oxytocin infusion became standard when establishing uterine contractions for induction, and remains effective unless the cervix is unripe [1,2]. By the 1970’s, prostaglandins (PGs) had been found effective cervical ripening and induction agents. Dinoprostone (PGE2) emerged as the preferred agent with commercial gels developed for vaginal [5] and intracervical [6] application, and more recently, slow release vaginal inserts [7]. Oral dinoprostone led to intolerable maternal gastrointestinal (GI) side effects, hence this approach was abandoned [8].

Misoprostol, a PGE1 analogue, was developed and marketed as an oral medication for prevention and treatment of nonsteroidal anti-inflammatory drug (NSAID) induced upper GI ulcers [9]. Pregnancy use was contraindicated due to its uterotonic effect. Because it was so inexpensive relative to PGE2 induction approved products, investigator driven research [10–13] for this purpose began in the early 1990’s. Found effective and seemingly safe in labor induction, misoprostol received widespread endorsement [1,2,14] for off label use. Misoprostol induces labor whether given by oral [15–17], buccal [18], sublingual [18] or vaginal [10–13,19] routes.

Contemporaneously, quality evidence has accumulated for effectiveness of transcervical balloon catheter placement in pre-induction ripening [20]. Mechanical ripening may be preferred from a safety perspective [21]. Recently a Dutch multicenter RCT (PROBAAT-II) concluded that oral misoprostol had similar safety and effectiveness to Foley catheter [22]. Oral misoprostol has been recommended as the cost-effective safest PG option [23–25], however uncertainty [25, 26] continues as to the optimal administration schedule.

This manuscript presents a three group RCT which was conducted to assess the effectiveness of oral misoprostol, low dose vaginal misoprostol and vaginal dinoprostone gel (the center’s established approach) for term labor induction, when the cervix was unripe.

Though portions of these results were presented at the Society of Maternal Fetal Medicine (SMFM) in New Orleans, Louisiana, in January 2002 [abstract #440; Am J Obstet Gynecol 2001;185(6):S203], and at the Society of Obstetricians and Gynaecologists of Canada Annual Clinical Meeting in Edmonton, Alberta, June 2004 [abstract # O-OBS-021; J Obstet Gynaecol Can 2004;26(5 suppl)S20], because of its ‘negative’ findings, this RCT was not submitted for peer reviewed publication. It has been noted on pages 27 and 80 in the Cochrane Library [17] as awaiting more data. Although this trial may now be considered dated, the induction approaches compared remain in active use, endorsed by the World Health Organization [14]. This RCT is methodologically sound, was rigorously conducted “stand alone” research, and provides relevant, valuable information with respect to labor induction today, which will contribute to systematic reviews [5,17,19].

An increase in interest and acceptability for labor induction by women and care-givers is anticipated [4]. The Cochrane Library [17] has proposed oral misoprostol as a preferred induction approach [23,24], although uncertainty [25,26] persists with respect to the actual regimen. This RCTs results support a readily administered oral misoprostol protocol, and raise concern regarding a low dose vaginal misoprostol protocol, very similar to that endorsed by WHO [14].

## Materials and methods

This study was conducted at the Izaak Walton Killam (IWK) Health Centre, Halifax, Nova Scotia, Canada, with subjects recruited between April 1, 1999 and December 31, 2000. Follow up was continued until postpartum discharge. Because subject enrollment began and was completed before July 1, 2005, this RCT has been registered retrospectively at www.clinicaltrials.gov. **Trial Registration**: NCT03489928. Registration was completed before manuscript submission. This process complies with the International Committee of Medical Journal Editors (ICJME) and PLOS ONE clinical trial registration policy. The authors have no ongoing and related trials, but will register future trials prospectively.

Annually, about 4500 of the province’s nine thousand births occur at this tertiary perinatal unit, which is the sole birthing center for a metropolitan population of approximately 400,000, serving a radius of 100 kilometers. The population is predominantly Caucasian. The research proposal was approved by the IWK Research Ethics Board (Project Number 1415, approved 9 November 1998)

Patients were eligible if they presented at greater than 37 weeks gestation with an indication for induction, and a single live cephalic fetus. Exclusion criteria were non-reassuring fetal heart rate (FHR) tracings, previous uterine surgery, known hypersensitivity to misoprostol or other PGs, uncontrolled epilepsy or asthma, or contraindication to vaginal birth. Randomization was stratified based on membrane status, intact or ruptured. Eligible subjects, who had given written informed consent, were assigned to an induction method by opening the next sequentially numbered opaque envelope for that stratum, only when induction was to begin. Envelopes contained a card indicating study allocation (oral misoprostol, vaginal misoprostol, or usual [vaginal dinoprostone]), prepared by an administrative staff member not involved in patient care, using computer generated random number tables with randomization in blocks of six and nine. Group assignment was concealed until the time of induction from patients and caregivers, who were unaware of the blocked randomization.

All study inductions were carried out on an inpatient basis, with continuous electronic monitoring (EFM) of FHR and uterine contractions for at least one hour after any PG administration. Patients randomized to the oral misoprostol (Cytotec; Searle Canada, Oakville, Ontario, Canada) group were initially given 50 μg of misoprostol orally (a 100 μg tablet pre-cut in half by pharmacy staff). This could be repeated at no sooner than four hour intervals until one of the following occurred: progressive labor, contraction frequency of three or more in 10 minutes, non-reassuring FHR tracing, or delivery. All decisions with regard to repeat misoprostol use, artificial membrane rupture, analgesia, epidural use, and oxytocin augmentation were made by the attending physician or a delegate, who reassessed the patient before each administration of oral misoprostol. Oxytocin augmentation was not permitted until four or more hours after discontinuing oral misoprostol. IV oxytocin began at two milliunits per minute and increased in increments of two milliunits every 30–60 minutes, as appropriate. Patients randomized to vaginal misoprostol had 25 μg (a 100μg tablet pre-cut in quarters by pharmacy staff) placed in the upper vagina. A repeat of this dose or an increase to 50 μg, based on caregiver assessment, could be given in no sooner than six hour intervals, under the provisos described above for oral misoprostol. Patients allocated to the vaginal dinoprostone protocol were managed according to our Health Centre’s established induction protocol: one or two mg of dinoprostone gel intravaginally (Prostin; Upjohn, Don Mills, Ontario, Canada) at no sooner than six hour intervals, if used, at the attending physician’s discretion, again under the provisos described above for oral misoprostol. Oxytocin augmentation was not permitted until more than six hours after vaginal PG.

The primary outcome measure was the time from induction at randomization to vaginal delivery. A 240-minute difference in means was chosen as clinically important based on response to a patient questionnaire before a prior study from this group [16]. Initial sample size calculation[27] was based on a two-tailed α = 0.05, power 95%, Δ = 240 minutes, and σ = 588 minutes (from the group’s prior published misoprostol RCTs [13,15, 16]). Twenty percent was then added to allow for anticipated cesarean births. For the primary outcome, this resulted in a study sample size (3N) of 510. The high power was chosen to increase confidence in a potential no difference result. Secondary outcomes addressed harm to the newborn (cord blood acidemia and birth asphyxia) and mother (cesareans and peripartum interventions), maternal GI intolerance, and excessive uterine activity.

No attempt was made at formal blinding of patients or caregivers following concealed randomization, although newborn assessment was carried out by a neonatal team unaware of study group assignment. All newborns were evaluated by Apgar score, cord blood acid-base analysis, and the recommended neurologic and general physical assessment [28]. A diagnosis of birth asphyxia required each of the following four findings: profound metabolic or mixed acidemia (cord artery pH less than 7.00), five minute Apgar score of three or less, neonatal neurologic abnormality, and dysfunction of one other major body system [28].

Maternal GI intolerance (nausea, vomiting, or diarrhea) was assessed by caregiver report and patient questionnaire. To determine the frequency of excessive uterine activity, all FHR tracings were reviewed by two of the investigators, blinded to study group and outcome. Differences were resolved by consensus. Using terminology defined by Curtis [29], tachysystole was considered a contraction frequency of more than five in a 10 minute period for two consecutive 10 minute periods, and hyperstimulation, the presence of tachysystole with late FHR decelerations, or other worrisome FHR changes. Other prespecified outcomes included labor intervals to vaginal birth (time to full dilation, and length of labor stages), frequency of maternal interventions and morbidity, birth method–vaginal (spontaneous, vacuum, or forceps) and cesarean, and frequency of vaginal birth within specified intervals following induction, such as within 24 hours (as encouraged by Cochrane [30]). Maternal satisfaction with labor was evaluated by questionnaire [31], which is provided in Supporting Information, S1 Study Protocol, pages 21–24.

Data on all subjects were analyzed in the group to which they were randomized (on an intent-to-treat basis) by parametric and non-parametric statistics, using Statistix 10.0 (Analytic Software, Tallahassee, FL). Decision levels and hypotheses to be tested were pre-set to minimize bias. Hypothesis testing was performed on the primary outcome. Other comparisons were considered hypothesis generating. Statistical significance for the primary outcome measure was assessed using analysis of variance (ANOVA) and was considered significant at P < 0.05. A rank order non-parametric analysis of the primary outcome using the Kruskal Wallis (KW) test was pre-specified, as it allowed inclusion of cesarean birth in vaginal birth outcomes, by ranking it longer than the longest achieved vaginal birth (as a failure to deliver vaginally) [13,15,16]. The sample size calculated for the parametric analysis would be appropriate for the non-parametric analysis [32]. Continuous variables were examined for normal distribution (Wilk-Shapiro test) before using parametric statistics. Non-parametric statistics are not compromised by skewed data distribution, and were used as appropriate. Pairwise differences in means and medians were evaluated by Tukey HSD (honestly significant difference) and Dunn tests respectively [33,34]. Categorical data were assessed with χ2 and Fisher Exact tests, as appropriate. Statistical significance for all secondary and hypothesis generating analyses was P < 0.001 to account for multiple testing, a conservative approach.

## Results

**Fig 1:**
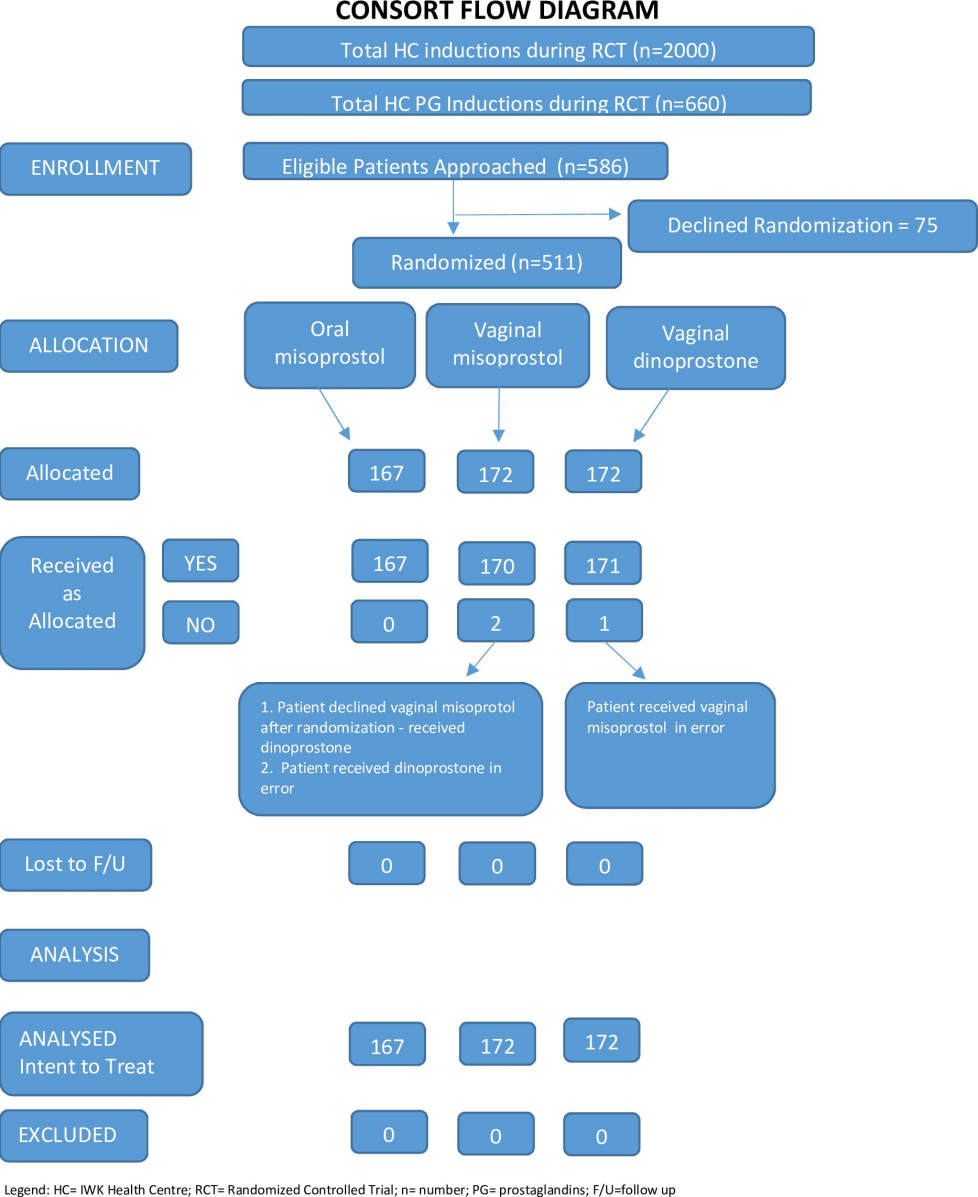
Consort flow diagram. HC = IWK Health Centre; RCT = Randomized Controlled Trial; n = number; PG = prostaglandins; CS = cesarean section (all low transverse). Two women with twins (ineligible) were randomized, one to vaginal misoprostol and one to vaginal dinoprostone. Maternal data are included. Only first twin of each pair is included in newborn data, but all four newborns had good outcomes after vaginal birth.

Participant flow is outlined in Fig 1. None of the 511 randomized patients were lost to follow-up. Maternal pre-induction and neonatal demographic data are presented in Table 1. The most common indications for induction were gestational age greater than 41 weeks (49.9%), hypertensive disorders (22.5%), and amniotic fluid concerns [PROM, or intact but largest fluid pocket less than two cm diameter] (12.3%), with no difference between the groups (χ2 = 3.55, P = 0.74). Peripartum data are given in Table 2. Though not reaching the pre-specified level for statistical significance in secondary outcomes, more subjects (Table 1) had Bishops’ score of seven or more in the vaginal dinoprostone group (P = 0.01), and more subjects (Table 2) used oxytocin infusions with oral misoprostol (P = 0.04)

**Table 1:** Demographics (Mean±SD).

	Oral Misoprostol (n = 167)	Vaginal Misoprostol (n = 172)	Vaginal Dinoprostone (n = 172)
Maternal Age (years)	29.1±6.6	28.8±5.6)	29.1±5.7
Weight (kg) *	89.1±18.8	89.4±17.1	88.5±16.0
Height (cm) *	162.7±7.6	162.4±6.6	162.3±6.9
Gestation (weeks)	40.0±1.5	39.4±1.4	40.0±1.5
Newborn Weight (g)	3574±523	3621±557	3598±530
Nullipara (n)	108 **64.7%**	107 **62.2%**	107 **62.2%**
Parity±SD	0.4±0.6	0.5±0.8	0.6±0.9
Bishop’s Score† ±SD	3.8±1.9	4.1±1.9	4.2±2.1
Bishop’s Score† < 7‡	145 **91.2%**	153 **91.6%**	138 **82.1%**
Membranes Intact	155 **92.8%**	155 **90.1%**	155 **90.1%**

SD-standard deviation; n-number; kg-kilogram; cm-centimeter; g-gram

*Weight was missing in 2, 5, and one subjects respectively, and height was missing in 71,79,and 78 subjects respectively

†-full Bishop Score components available on 159, 167, and 168 subjects respectively

‡ P = 0.01; Percentages for categorical data are **bolded**

**Table 2:** Peripartum data, adverse effects, and maternal satisfaction.

	Oral Misoprostol (n = 167)	Vaginal Misoprostol (n = 172)	Vaginal Dinoprostone (n = 172)	P
***Intrapartum Frequency***				
Oxytocin Used	117 **70.0%**	98 **57.0%**	113 **65.7%**	0.04
Epidural	90 **53.9%**	89 **51.7%**	101 **58.7%**	0.6
No Analgesia	12 **7.2%**	14 **8.1%**	13 **7.6%**	0.95
Meconium	37 **22.2%**	39 **22.7%**	45 **26.2%**	0.62
Scalp pH	12 **7.2%**	18 **10.5%**	14 **8.1%**	0.58
***Perineal Trauma***				
Episiotomy	30 **18.0%**	36 **20.9%**	27 **15.7%**	0.45
3rd /4th tears	5 **3.0%**	7 **4.1%**	3 **1.7%**	0.4
Intact	79 **47.3%**	70 **40.7%**	62 **36.1%**	0.11
**Adverse Effects**				
***GI Effects***				
Diarrhea	0	3 **1.7%**	4 **2.3%**	0.16
Nausea	16 **9.6%**	16 **9.3%**	20 **11.6%**	0.73
Vomiting	29 **17.4%**	24 **14.0%**	37 **21.5%**	0.18
***Excess Uterine Activity***				
Tachysystole*	36 **21.7%**	51 **29.8%**	40 **23.4%**	0.19
Hyperstimulation†	8 **4.8%**	16 **9.4%**	12 **7.0%**	0.27
**Maternal Satisfaction**				
	n = 145	n = 139	n = 139	
*Want in future induction*	112 **77.2%**	108 **77.7%**	102 **73.4%**	0.86
Sense of Control**	n = 137	n = 130	n = 146	
Mean ±SD	99.7 ±18.2	99.5 ±19.3	103.5 ±15.8	0.11
Median (Q1-3)	101 (88–114)	104 (85–115)	105.5 (93.8–116)	0.22

n-number; P-probability; Scalp pH-fetal scalp blood sampled for pH; Percentages for categorical data are **bolded;**

*two consecutive 10 minute windows

†tachysystole plus non-reassuring FHR; under **Maternal Satisfaction**, n-number of respondents; *Want in future induction*-yes response to the question “If you needed a labor induction in another pregnancy, would you want to have the same induction method”

** *Sense of Control*–a total score of each subject was obtained by reversing the scores of positively worded items (7 = 1, 6 = 2, etc) and then summing the items; SD-standard deviation; Q-quartile

Time to vaginal birth intervals are presented in Table 3. The mean time from randomization and induction to vaginal birth, the primary outcome, identified no significant difference between the three groups (ANOVA, F = 0.79, df = 2/352, P = 0.45). No pairwise differences [33] in means between groups were significant (Tukey HSD). The standard deviation in the vaginal misoprostol group for this outcome, as well as that for time to full dilation, was quite different from that in the other two groups. Both these intervals include pre-induction cervical ripening time. In three subjects more than a two day rest break occurred during cervical ripening. All were parous and in the vaginal misoprostol group. Times from induction to vaginal birth were respectively: more than 22 days (initial induction indication PROM subsequently revised with induction deferred for 21 days), ten days (rest of one week after six doses of misoprostol without labor onset, or fetal concerns), and five days (cephalic to breech spontaneous version after induction began hence induction deferral, then spontaneous version back to cephalic with induction restarted). All three resulted in spontaneous vaginal delivery with good newborn outcomes. The Shapiro-Wilk test indicates substantial deviation of the time to vaginal delivery data from a normal distribution (W = 0.2855, P (W) < 0.0001, 355 cases). There was little difference between the groups in lengths of the first (P = 0.44) and second (P = 0.72) stages, after active labor was established.

**Table 3:** Labor intervals to vaginal births (in minutes) and newborn outcomes.

	Oral Misoprostol	Vaginal Misoprostol	Vaginal Dinoprostone	P
***Parametric Analysis N*** *Mean±SD*	112	118	125	
Induction To Vaginal Birth	1356±1033	1530±3249	1208±613	0.46
To 10 cm	1261±1014	1437±3257	1123±598	0.47
1st Stage	402±331	354±248	372±272	0.44
2nd Stage	94±94	93±104	85±83	0.72
***Nonparametric Analysis N***	167	172	172	
Median (Q1-3)	1571 (883-CS)	1338.5 (825-CS)	1451.5 (915-CS)	0.45
Vaginal births N<72 h	109 **65.3%**	115 **66.9%**	125 **72.7%**	0.30
N<48 h	107 **64.1%**	115 **66.9%**	122 **70.9%**	0.40
N < 24 h	75 **44.9%**	92 **53.5%**	82 **47.7%**	0.27
N< 12 h	23 **13.8%**	30 **17.4%**	26 **15.1%**	0.64
***Birth Method***				
SVD	88 **52.7%**	94 **54.7%**	110 **64.0%**	
Vacuum	4 **2.4%**	5 **2.9%**	5 **2.9%**	
Forceps	20 **12.0%**	19 **11.0%**	10 **5.8%**	
Cesarean	55 **32.9%**	54 **31.4%**	47 **27.3%**	0.21*
**Newborn Outcomes**				
Mean pH^†^ (SD)	7.25±0.07	7.24±0.09	7.25±0.08	0.33
Mean Base^†^ Deficit^‡^ ±SD	3.4±3.1	4.0±3.5	3.7±3.3	0.35
Median Apgar^1min^ (Q1-3)	9 (8–9)	9 (7–9)	9 (8–9)	0.15
# Apgar^1min^ < 7	27 **16.2%**	37 **21.5%**	24 **14.0%**	0.16
Median Apgar^5min^ (Q1-3)	9 (9–10)	9 (9–10)	9 (9–10)	0.74
# Apgar^5min^ < 7	4 **2.4%**	3 **1.7%**	2 **1.2%**	0.69
***Birth Asphyxia Criteria***				
Apgar^5min^ < 4	0	2 **1.2%**	0	0.33^¶^
Arterial pH^†^ < 7.0	0	4 **2.7%**	0	0.04^¶^
Base Deficit^†^ N>16.0^‡^	0	1 **0.7%**	0	1.00^¶^

P-probability; N-number; SD-standard deviation); cm-centimeter; Q-quartile; CS-cesarean; h-hours; SVD-spontaneous vaginal delivery; Percentages for categorical data are **bolded**

*-Birth method P is for 3 by 3 Table with vacuum and forceps merged as operative vaginal birth, χ^2^ = 5.848

^†^-Acid-base data is for 132, 150, and 129 subjects respectively

^‡^-mmol/L ^¶^-Where expected numbers<5, Freeman Halton extension of Fisher Exact used.

The non-parametric analysis of vaginal birth intervals is also given in Table 3. No difference of statistical significance was found in median times from randomization and induction to vaginal birth between the groups (KW, F = 0.79, df = 2/508, P = 0.45). No pairwise differences [34] in ranks between groups were found (Dunn). Birth method is shown in Table 3. There was no difference in rate of cesarean section versus vaginal birth (χ2 = 7.14, P = 0.31). Neither Kaplan-Meier survival analyses for time to vaginal birth with time to cesarean birth considered censored (logrank test χ2 = 4.57,df = 2,P = 0.10), nor with time to cesarean ranked longer than the longest achieved vaginal birth (logrank test χ2 = 1.47,df = 2. P = 0.48) were significantly different. The survival curves are provided in Supporting Information S1 Fig and S2 Fig respectively.

Table 4 and Table 5 give analyses via parametric and non-parametric statistics for women separated as nulliparous and parous for the time to vaginal birth outcome. Sensitivity analyses for overall and parity based comparisons are given: with removal of all births more than five days after randomization (hence eliminating the effect of the three outliers with long rest breaks in the parous vaginal misoprostol group), and more than four days after randomization (consistent with the approach in PROBAAT-II [22]). None of the non-parametric statistics reveal differences at even the P<0.05 level. Median time to vaginal birth is above 1800 minutes in every nulliparous group and below 1200 minutes in every parous analysis (P<0.0001, Wilcoxon Rank Sum). By 48 hours, 97.2% of nulliparous and 96.6% of parous subjects who ever achieved a vaginal birth, had done so, compared with 63.0% and 77.6% respectively at 24 hours (P<0.0001, χ2)(Table 6). Sensitivity analyses (Table 4) demonstrate the influence of the few rest break outliers on parametric (ANOVA) statistics. Secondary analyses by membrane status, intact or ruptured, is provided in Table 7. No significant differences were found.

**Table 4:** Parametric analyses induction intervals to vaginal birth by parity (in minutes).

	Oral Misoprostol		Vaginal Misoprostol		Vaginal Dinoprostone		P
*Primary Outcome*	N	Mean±SD	N	Mean±SD	N	Mean±SD	
Overall	112	1356±1033	118	1530±3249	125	1208±613	0.46
Nulliparous*	58	1609±953	58	1223±501	65	1286±659	0.01
Parous	54	1083±1055	60	1828±4528	60	1123±552	0.26
***Sensitivity Analysis-Births>7200 min (5 days) removed***							
Overall	112	1356±1053	115	1087±531	125	1208±613	0.03
Nulliparous*	58	1609±953	58	1223±501	65	1286±659	0.01
Parous	54	1083±1055	57	948±529	60	1123±552	0.42
***Sensitivity Analysis***-***Births> 5760 min (4 days) removed***							
Overall	110	1255±729	115	1087±531	125	1208±613	0.11
Nulliparous*	57	1514±618	58	1223±501	65	1286±659	0.02
Parous	53	980±743	57	948±529	60	1123±552	0.26

P-Probability; N-number; SD-standard deviation; min-minutes

*No pairwise differences (Tukey HSD) at P = 0.01; Data from each row deviates from a normal distribution (Shapiro Wilk Normality Test W>0.75, P<0.0001)

**Table 5:** Nonparametric analyses induction intervals to vaginal birth by parity (in minutes).

	Oral Misoprostol		Vaginal Misoprostol		Vaginal Dinoprostone		P
	N	Median(Q_1_-Q_3_)	N	Median(Q_1_-Q_3_)	N	Median(Q_1_-Q_3_)	
Overall	167	1571 (883-CS)	172	1338.5 (825-CS)	172	1451.5 (915-CS)	0.45
Nulliparous	108	2696 (1420.3-CS)	107	1897 (1146-CS)	107	1918 (964-CS)	0.11
Parous	59	877 (584–1396)	65	891 (586.5–1371)	65	1164 (764.5–1557.5)	0.15
***SensitivityAnalysis-Births>7200 min (5 days) removed***							
Overall	165	1564 (882-CS)	168	1325.5 (822.8-CS)	172	1451.5(915-CS)	0.45
Nulliparous	106	2610.5 (1417.5-CS)	106	1849.5 (1145-CS)	107	1918 (964-CS)	0.15
Parous	59	877 (584–1396)	62	841.5 (580–1309)	65	1164 (764.5–1557.5)	0.09
***SensitivityAnalysis***-***Births>5760 min (4 days) removed***							
Overall	163	1542 (881-CS)	167	1319 (822-CS)	172	1451.5(915-CS)	0.47
Nulliparous	105	2589 (1415-CS)	105	1802 (1144-CS)	107	1918 (964-CS)	0.14
Parous	58	873.5 (584–1361.5)	62	841.5 (580–1309)	65	1164 (764.5–1557.5)	0.08

P-Probability; N-number; CS-cesarean; Q_1_,Q_3_-quartiles; min-minutes

**Table 6:** Vaginal births achieved within specific times, by parity.

Nulliparous					Parous			
	Oral Misoprostol	Vaginal Misoprostol	Vaginal Dinoprostone	P	Oral Misoprostol	Vaginal Misoprostol	Vaginal Dinoprostone	P
N	108	107	107		59	65	65	
Vaginal births N<12 h	3 **2.8%**	9 **8.4%**	11 **20.3%**	0.08	20 **33.9%**	21 **32.3%**	15 **23.1%**	0.35
N<24 h	30 **27.8%**	42 **39.3%**	42 **39.3%**	0.13	45 **76.3%**	50 **76.9%**	40 **61.5%**	0.09
N<48 h	55 **50.9%**	58 **54.2%**	63 **58.9%**	0.50	52 **88.1%**	57 **87.7%**	59 **90.8%**	0.83
N<72 h	57 **52.8%**	58 **54.2%**	65 **60.8%**	0.46	52 **88.1%**	57 **87.7%**	60 **92.3%**	0.64
Ever	58 **53.7%**	58 **54.2%**	65 **60.8%**	0.51	54 **91.5%**	60 **92.3%**	60 **92.3%**	0.98

P-Probability; N-number; h-hours; Percentages for categorical data are **bolded**

**Table 7:** Labor intervals to vaginal birth by membrane status.

**Membranes Intact**		**Oral Misoprostol**		**Vaginal Misoprostol**		**Vaginal Dinoprostone**		**P**
Parametric Analysis								
N		103		106		111		
Mean ±SD min		1370±1067		1326±1619		1262±619		0.79
Non-Parametric Analysis								
N		155		155		155		
Median (Q_1_-Q_3_) min		1573 (881-CS)		1385 (873-CS)		1504 (945-CS)		0.72
Vaginal Births								
N	<72h	100	64.5%	104	67.1%	111	71.6%	0.40
	<48h	98	63.2%	104	67.1%	108	69.7%	0.48
	<24h	69	44.5%	21	52.3%	74	47.7%	0.29
	<12h	21	13.5%	24	15.5%	24	15.5%	0.85
Caesareans		52	33.5%	49	31.6%	44	28.4%	0.61
**Membranes Ruptured**		**Oral Misoprostol**		**Vaginal Misoprostol**		**Vaginal Dinoprostone**		**P**
Parametric Analysis								
N		9		12		14		
Mean ±SD min		1184±514		3338±9126		776±341		0.46
Non-Parametric Analysis								
N		12		17		17		
Median (Q_1_-Q_3_) min		1435 (1213–37936)		822 (687-CS)		827 (601–1310)		0.22
Vaginal Births								
N	<72h	9	75.0%	11	64.7%	14	82.4%	0.50
	<48h	9	75.0%	11	64.7%	14	82.4%	0.50
	<24h	6	50.0%	11	64.7%	13	76.5%	0.34
	<12h	2	16.7%	6	35.3%	5	29.4%	0.54
Caesareans		3	25.0%	5	29.4%	3	17.6%	0.72

P-Probability; N-number: SD-standard deviation; min-minutes; Q_1_,Q_3_-quartiles; CS-caesarean section; h-hours; No pairwise differences for means or medians were significantly different (P>0.05) (Tukey HSD or Dunn Test, as appropriate)

Maternal GI side-effects and excessive uterine activity results are given in Table 2. No differences were identified. Table 3 gives various newborn outcomes. No differences reached the pre-set limits for statistical significance. However, five minute Apgar score of three or less, and cord acid base measures of profound mixed or metabolic academia [28] were only encountered after vaginal misoprostol. With respect to cord pH less than 7.0, four occurred (P = 0.04, Fisher exact).

There were no maternal or neonatal deaths. A single newborn met the birth asphyxia definition [28]. Three vaginal misoprostol doses of 25, 50 and 25 μg in succession had been administered as per protocol. No uterine hyperstimulation was identified. Birth was by cesarean after unsuccessful trial of forceps more than 30 hours after the last dose of misoprostol. Five minute Apgar score was three, cord artery pH 6.86 and base deficit 18.0 mmol/L. The newborn was intubated for ventilatory support. Seizures were treated with anticonvulsants. Neuroimaging was normal.

An additional three newborns had a seizure: two who were induced with vaginal misoprostol (hypoglycemia in one, and small occipital cerebral infarct in the other), and one who received oral misoprostol (small intracranial bleed after forceps). Neither of these events was associated with birth acidemia or low Apgar. Meconium aspiration syndrome and/or pneumothorax were diagnosed in three newborns induced with each of vaginal or oral misoprostol, and four from the vaginal dinoprostone group.

The only significant maternal morbidity was one postpartum hemorrhage in each group, where the maternal hematocrit had an absolute fall greater than 15%. Only the mother who was induced with vaginal misoprostol received blood products (five units each of packed red cells and fresh frozen plasma). She also underwent hysterectomy. A para two, she had had an uncomplicated induction with 25 μg vaginal misoprostol, followed in six hours with 50 μg vaginally. She had a spontaneous vaginal birth of healthy 2809 g newborn without oxytocin augmentation, after a 148 min first and 103 minute second stage of labor.

No differences were found between the groups in maternal satisfaction or sense of control [31] in labor (KW, F = 1.53, df = 2/410, P = 0.22) as shown in Table 2. To the specific question “if you needed a labor induction in another pregnancy, would you want to have the same induction method?” 76% of the 423 who responded would choose the same method of induction, if needed it in the future, with no difference between methods (χ2 = 0.86, P = 0.65).

Although a median of two doses of any prostaglandin was used in each group, fewer overall doses were used with vaginal misoprostol and vaginal dinoprostone, than with oral misoprostol (Dunn pairwise, KW, F = 22.48, df = 2/508 P<0.001). Maximum PG doses used were eleven for each of oral and vaginal misoprostol, and six for the vaginal dinoprostone approach. Ninety seven women (56.4%) receiving vaginal misoprostol used only 25 μg doses.

## Discussion

There were no significant differences in the effectiveness of oral misoprostol (50 μg po q4h prn), low dose vaginal misoprostol, or the center’s established induction protocol, using industry prepared vaginal dinoprostone gel. This is consistent with available systematic reviews [17,19,23,24]. Parity has an obvious influence on induction duration and outcome measures. There was no difference in maternal satisfaction, GI side effects, peripartum interventions, perineal trauma, or birth route between the induction approaches. No difference was found in frequency of excessive uterine activity or fetal scalp blood sampling use. Clinicians can make their choice of induction agent depending on cost, local logistics, and patient preference. From the broad perspective, this will allow clinicians to avoid vaginal administration in those with previous sexual abuse, avoid misoprostol in units whose managers have regulatory concerns or pharmacy has difficulty providing accurately cut tablets, and avoid dinoprostone in those units where there is difficulty with drug refrigeration.

Vaginal misoprostol was used in the low dose (25 μg) and at the widest interval (no sooner than every six hours) currently endorsed by national [1,2] and WHO international [14] guidelines. Though not reaching the pre-specified, but conservative, significance level, there was nevertheless a worrisome frequency of profound newborn acidemia (cord artery pH <7.0) in vaginal misoprostol subjects (P = 0.04, Fisher Exact). No subject receiving oral misoprostol or vaginal dinoprostone induction had this outcome.

That this RCT was conducted in 1999–2000 may be considered a limitation. Have changes in patient demographics or induction practice occurred since then which preclude relevance? A recent national conference presentation with published abstract [35], and Nova Scotia Reproductive Care Program perinatal indicator reports [36,37] provide insight on the regional Nova Scotian maternal demographics in 2000, versus today. Maternal age and pre-pregnancy weight have increased. Proportion of nulliparous births has remained close to 45% with no obvious trend. Use of intrapartum epidural and assisted vaginal birth have not changed substantially. Notably, labor induction rate has increased from 25% to 33%, and cesarean section from 23% to 28% over that time. The most frequent induction indications remain post-term pregnancy, pregnancy hypertension and PROM. Importantly, the labor induction protocols compared herein continue in active use and endorsed today [1–3,14]. Taken together, despite some changes in maternal demographics that might reduce induction effectiveness, the stability of indications, consistent parity proportion, as well as continuing use and endorsement of methods studied, support the relevance and generalizability of these results to the current Nova Scotia population. The demographic data and analyses by parity and membrane status provided permit readers in another setting to make their own decisions on this. Uncertainty remains as to the preferred induction approach [17,25,26]. The observed increase in labor induction and cesareans highlights the importance of methodologically sound research on this topic.

This RCT was carried out in a rigorous “intent to treat” manner, strengthening the study’s internal validity. All randomized subjects were analyzed as assigned. As such, however, another issue might be that three subjects were major outliers with prolonged time to vaginal delivery. There was no pre-specified arbitrary “failed induction” definition [38]. Subjects were not withdrawn if a caregiver chose to recommend a rest period during the induction process. Such occurrences were “real life” events that enhanced generalizability. In all, nine subjects (1.8%) remained undelivered four days after randomization (2.4% of those randomized to oral misoprostol, 2.9% of those to vaginal misoprostol, and none to dinoprostone); as compared with the PROBAAT-II experience [22] of 6.4% for oral misoprostol, and 2.5% for Foley. Two subjects who received oral misoprostol, and the three “rest break” subjects discussed above who received vaginal misoprostol went on to vaginal birth. These outlier vaginal births were responsible, in large part, for the substantial unexpected increase in standard deviation observed in the oral misoprostol and vaginal misoprostol groups in the primary outcome parametric analysis compared to the figure used in sample size calculation. Table 3 parametric analysis data indicate that the issue arose during the prelabor ripening prior to active labor (first and second stages). Effect size (difference in group means divided by standard deviation) used in sample size computation was therefore not realized. Looking at the sensitivity analysis overall data in Table 4, shows that with induction to vaginal birth more than 5760 minutes (four days) removed, standard deviations are very similar to the assumptions used in sample size calculation. Power for the planned primary outcome parametric analysis was reduced substantially by the outlier vaginal births.

The preplanned non-parametric cesarean ranked longest [13,15,16] analysis of time to vaginal birth allowed inclusion of primary outcome data on all subjects. Even in a survival analysis of labor lengths, a cesarean is more appropriately handled in this manner, rather than considered simply as censored [32]. The birth outcome is known. A cesarean may not be independent of induction method. Also, the non-parametric approach is robust to the presence of outliers [36], the non-normal distribution of the time to vaginal birth data, and loses little or no power [32,36]. While recognizing that safe vaginal birth is the ideal primary outcome in RCTs assessing induction success, the non-parametric cesareans ranked longest analysis comparing times to vaginal birth with medians as the measure of central tendency, served as a better surrogate than the more frequently chosen parametric analysis with means.

This RCT includes data on all five primary outcomes recommended in the Cochrane Library generic protocol for cervical ripening and induction [30]. In addition, data are reported on more than 80% of the other secondary outcomes in Cochrane [17,30]. The largest impact of adding this manuscript’s results would be in meta-analyses of 50 μg oral misoprostol versus vaginal dinoprostone where any Cochrane meta-analysis includes four or fewer RCTs. All these meta-analyses but one have fewer than 700 subjects in total [17]. Cochrane authors specifically request information on adverse events and serious newborn and maternal morbidity/mortality, which are provided herein. A patient satisfaction survey is reported, which has infrequently been conducted in prior studies. No RCTs in the Cochrane Library using oral misoprostol versus vaginal dinoprostone and only one of oral misoprostol versus vaginal misoprostol, including 204 subjects, have reported on maternal satisfaction [17]. Cochrane encourages use of vaginal birth not achieved in 24 hours as a primary outcome [30]. Others [22] feel this is problematic as it discounts successful, though longer duration, vaginal births. This RCTs results show substantial gains in vaginal birth up to 48 hours, which is also captured by the cesareans ranked longest nonparametric analysis.

Prior PG pharmacokinetic research [39–43] provides a rationale for the findings presented. Though peak serum levels are reached at 70–80 minutes after administration, studies with vaginal misoprostol have found detectable, even increasing and clinically relevant misoprostol acid levels after a single dose until the time limit studied, six hours [40]. Clinicians often find misoprostol tablet remnants from prior doses on a subsequent vaginal exam [41]. In addition, as with other vaginally administered drugs, uterine effects may reflect local tissue levels from direct transfer [42], and may not be represented by serum levels. Uterine activity in Montevideo units (MU) after a single dose of vaginal misoprostol has continued to increase four hours after placement, the duration of study [43]. This all raises the concern of cumulative effects with additional doses of vaginal misoprostol within this time interval.

Oral misoprostol reaches peak levels in 30 minutes, falling to 10% of peak levels at two hours, and just detectable at four hours [40]. The half time is 20–40 minutes. Recommended dosing schedule for its marketed GI indication is every six hours [9]. Uterine activity after a single dose peaked at one hour, remaining at that level without rising for the duration of the four hours studied [43]. This RCTs oral misoprostol dose of 50 μg, up to every four hours as needed was not different in effectiveness or safety measures than the vaginal dinoprostone protocol. The Dutch multicenter RCT (PROBAAT-II) found this regimen of oral misoprostol neither less effective nor less safe than Foley catheter [22].

Oral misoprostol protocols [25], with lower though titrated doses, but at intervals less than four hours, strive to be more effective, but may be nearing the cumulative effect threshold for oral administration, with the concern of adverse newborn effects that may be heralded by uterine tachysystole and worrisome FHR changes. The recent Indian RCT (INFORM) of women induced for hypertension found oral misoprostol, 25 μg every two hours, more effective than Foley [44]. Nulliparous subjects, who made up 80% of those randomized, were eligible to increase their oral misoprostol to 50 μg q2h after two doses, if needed. Though listed as a secondary outcome, the frequency of this dose increase was not reported in the published manuscript [44] nor online appendix. Rates of tachysystole and FHR changes were evaluated by intermittent auscultation and clinical exam, not EFM. No increase in newborn morbidity was noted in the 302 subjects randomized to oral misoprostol. Administering misoprostol orally 50 μg every two hours, is aggressive, and begs greater scrutiny, before acceptance into practice.

There is a void [17,25,26] and urgent need for direct “head to head” comparisons of oral misoprostol regimens for labor induction, an obvious example being 50 μg q4h of this report versus 25 μg q2h. These should be well designed RCTs with sufficient power to address meaningful outcomes [maternal/effectiveness (cesarean rates) and newborn/safety (like those herein)]. Oral protocols seem more amenable to masking, a design strength.

Clinical trials have been completed with a slow release misoprostol vaginal insert(MVI), now marketed in Europe [45]. Despite having comparable effectiveness [46] to the dinoprostone slow release vaginal system (DVI), the misoprostol device had a higher removal rate for adverse events, including FHR concerns [46,47]. This outcome is consistent with misoprostol’s pharmacokinetics described above, relative to dinoprostone’s (half time three minutes [47]). Thus the anticipated safety benefit of MVI quick removal remains to be shown [48,49]. Even without this concern, a commercially marketed MVIs pricing will likely remain unattractive relative to oral misoprostol.

## Conclusions

If time to vaginal birth is the chosen effectiveness outcome in labor induction, non-parametric cesareans ranked longest analysis is recommended.

No statistically significant difference was found in outcomes between oral misoprostol (50 μg po q4h prn), low dose vaginal misoprostol, or the established dinoprostone vaginal gel protocol for cervical ripening and induction. Clinicians can choose among these induction agents depending on cost, local logistics, and patient preference. Profound newborn acidemia, though infrequent, was encountered only with low dose vaginal misoprostol.

## Supporting information

S1 CONSORT Checklist CONSORT 2010 checklist of information to include when reporting a randomised trial*. (DOC)

S1 Study Protocol (PDF)

S1 Data Dictionary Database variables for misoprostol RCT PLOS 2019. (PDF)

S1 Fig Kaplan-Meier survivorship function curve for time to vaginal birth with cesarean censored. Y-axis: proportion of subjects not yet with vaginal birth. X-axis: time since induction begun (minutes). Time variable: TIMTODEL-time to vaginal birth. Cesarean births are censored. Event variable: VagBir-vaginal birth. Plot is truncated at 5760 minutes (4 days). Hence, for oral misoprostol, 2 vaginal births and 2 cesareans, and, for vaginal misorpostol, 3 vaginal births and 2 cesareans are not represented.
(TIF)

S2 Fig Kaplan-Meier survivorship function curve for time to vaginal birth with cesareans ranked longest. Y-axis: proportion of subjects not yet with vaginal birth. X-axis: time since induction begun (minutes). Time variable: TIMRANK-time to vaginal birth. Cesarean births ranked longest Event variable: VagBir-vaginal birth. Plot is truncated at 5760 minutes (4 days). Hence, for oral misoprostol, 2 vaginal births, and, for vaginal misoprostol, 3 vaginal births are not represented.
(TIF)
