# Correction: Oral misoprostol, low dose vaginal misoprostol, and vaginal dinoprostone for labor induction: Randomized controlled trial

**DOI:** 10.1371/journal.pone.0304233

**Published:** 2024-05-20

**Authors:** David C. Young, Tina Delaney, B. Anthony Armson, Cora Fanning

There are errors in Table 7. The values in vaginal births <24h and <12h under Membranes Intact are incorrect. The authors have provided corrected version here.

**Table 7:** Labor intervals to vaginal birth by membrane status.

Membranes Intact		Oral Misoprostol		Vaginal Misoprostol		Vaginal Dinoprostone		P
Parametric Analysis N		103		106		111		
Mean ±SD min		1370±1067		1326±1619		1262±619		0.79
Non-Parametric Analysis N		155		155		155		
Median (Q1-Q3) min		1573 (881-CS)		1385 (873-CS)		1504 (945-CS)		0.72
Vaginal Births N	<72h	100	64.5%	104	67.1%	111	71.6%	0.40
	<48h	98	63.2%	104	67.1%	108	69.7%	0.48
	<24h	69	44.5%	81	52.3%	69	44.5%	0.29
	<12h	21	13.5%	24	15.5%	21	13.5%	0.85
Caesareans		52	33.5%	49	31.6%	44	28.4%	0.61
**Membranes Ruptured**		**Oral Misoprostol**		**Vaginal Misoprostol**		**Vaginal Dinoprostone**		**P**
Parametric Analysis N		9		12		14		
Mean ±SD min		1184±514		3338±9126		776±341		0.46
Non-Parametric Analysis N		12		17		17		
Median (Q1-Q3) min		1435 (1213–37936)		822 (687-CS)		827 (601–1310)		0.22
Vaginal Births N	<72h	9	75.0%	11	64.7%	14	82.4%	0.50
	<48h	9	75.0%	11	64.7%	14	82.4%	0.50
	<24h	6	50.0%	11	64.7%	13	76.5%	0.34
	<12h	2	16.7%	6	35.3%	5	29.4%	0.54
Caesareans		3	25.0%	5	29.4%	3	17.6%	0.72
