# Supplementary material for: Oral misoprostol, low dose vaginal misoprostol, and vaginal dinoprostone for labor induction: Randomized controlled trial
Source: PLoS One. 2020 Jan 10;15(1):e0227245. doi: 10.1371/journal.pone.0227245 (PMC6953875; doi:10.1371/journal.pone.0227245)
Supplement: S1 Study Protocol — (PDF) [file pone.0227245.s002.pdf]

S2_ Study Protocol: Content Sections.. page 1

Study Consent Form……………………………………………………………………………… page 2-4
Consent Form. Misoprostol Labour Induction Study

RCT Outline……………………………………………………………………………………………. page 5
Randomized Control Trial of Women with Indication for Induction of Labor

Information distributed to care givers introducing RCT……………………………… page 6
Misoprostol Labour Induction Study

REB Submission Summary ……………………………………………………………………….. page 7-10
Research Summary – Misoprostol Labour Induction Study

RCT Protocol submitted to IWK Research Services………………………………………….. pages 11-17
Category B grant application – Misoprostol Labour Induction Study

Study Data Collection Sheet………………………………………………………………………….. page 18
Misoprostol Labor Induction Study

Birth Data Collection Sheet…………………………………………………………………………… page 19
Birth Record

Uterine Hyperstimulation Laminated Card distributed to all care givers……………… page 20
Uterine Hyperstimulation Protocol for Medical Management

Satisfaction and GI side effects questionnaire……………………………………………… Page 21-24
Misoprostol Labour Induction Study Questionnaire

CONSENT FORM

IWK Grace
HEALTH CENTRE

IWK Grace Health Centre for Children, Women & Families
5850/5980 University Avenue, PO Box 3070
Halifax, Nova Scotia, Canada B3J 3G9
Tel: (902) 428-8888

Study Title: Misoprostol Labour Induction Study **Investigator:** Dr. David C. Young

Study Site: Labour and Delivery
IWK Grace Health Centre
5850/5980 University Avenue
P. O. Box 3070, Halifax, NS
B3J 3G9

Sponsor: No external funding

INTRODUCTION: You are being asked to participate in a clinical research study. You understand that you have a reason for induction of labour and that your induction has been scheduled. Our traditional medical method of labour induction uses an intravenous drip with a hormone called oxytocin which produces uterine contractions, and vaginal placement of gel called dinoprostone, a prostaglandin, which produces uterine contractions but also softens and prepares the cervix for labour. We wish to study a different prostaglandin, misoprostol, in labour induction.

PURPOSE OF STUDY: Misoprostol is marketed for the prevention and treatment of stomach ulcers. It is made as a tablet to be taken by mouth, but can also be placed in the vagina. Recent research has shown that using misoprostol in either way will soften the cervix, begin uterine contractions, and induce labour. The purpose of this study is to compare misoprostol tablets taken by mouth, or placed in the vagina, with our usual induction methods. It is hoped that this research may result in labour induction being more natural and comfortable.

STUDY DESIGN: This is a randomized controlled trial similar to others that have been undertaken at several centres across Canada and worldwide. We hope to enroll 510 pregnant women needing induction of labour from the IWK Grace Health Centre.

Study Title: Misoprostol Labour Induction Study

SCREENING FOR YOUR PARTICIPATION: Before asking you to participate in this study, your doctor and the study coordinator or member of the study team will make sure you are eligible for this study. You must have a reason for which your physician recommends induction of labour, and there is a need for medications to soften your cervix or to initiate contractions. You should not participate in this study if you are allergic to misoprostol or other prostaglandins, or have uncontrolled asthma or epilepsy.

Participation in this study will begin with the induction of your labour, and end when you leave hospital following the birth of your baby.Your participation is entirely voluntary. If you decide not to participate, or to withdraw from of this study, your medical care at the IWK Grace Health Centre will not be affected in any way.

STUDY PROCEDURES: If you agree to become a participant, you will be randomly assigned or chosen by chance, like flipping a coin, to receive your induction medication in one of three ways. One group of patients will receive a portion of a misoprostol tablet by mouth, a second group of patients will have a portion of a misoprostol tablet placed in the vagina, and the final group will have labour induction by our usual method (a combination of dinoprostone gel placed in the cervix or vagina, and oxytocin given by an intravenous line as your doctor feels appropriate).

Your attending physician will continue to be responsible for all other aspects of your care. There are no additional blood tests or examinations, other than for the placement of medications. After the birth of your baby, your chart will be reviewed by a member of the research team for information regarding your labour, delivery, and your baby. Prior to your hospital discharge, we will be asking you to complete a brief (approximately 15 minutes) questionnaire on your satisfaction with your induction and birthing experience. You may choose to withdraw from this study at any time.

RISKS AND DISCOMFORTS: The risks of this study, in addition to those of induction of labour, are those associated with ingestion or insertion of misoprostol. In the low dosage used in labour induction, gastrointestinal upset is rarely present and, if so, is mild and of short duration. Nausea, vomiting, headache, diarrhea, or constipation have not been found any more frequently than with other labour inductions. As with any induction of labour, should your contractions occur more strongly or frequently than desirable, such that an adverse effect on your baby might occur (reducing oxygen supply through the placenta), the medication might have to be stopped, or removed (tablets or gel in the vagina). Rarely, a drug (ritodrine) may be given intravenously under such circumstances to relax the uterus. This approach would be necessary for misoprostol given by mouth.

POSSIBLE BENEFITS: You may experience no direct benefit. The information obtained from this study may help other pregnant women who need an induction of labour in the future, by providing more choices in approach.

Study Title: Misoprostol Labour Induction Study

ALTERNATIVE TREATMENTS: The alternative, should you choose not to enter the study, would be the usual management at our centre - vaginal application of dinoprostone gel, and intravenous oxytocin drip.

COMPENSATION: There will be no costs to you for being in the study. You will not be charged for research drugs or any research procedures.

CONFIDENTIALITY: Portions of your confidential hospital records directly relevant to this study will be reviewed by a study investigator.

Any information collected will be coded to protect your privacy. Should results of this study be published, you will not be identified. Study files will be kept in a locked filing cabinet. As part of an audit of research practices in this hospital, a representative of the Research Office and/or the Health Protection Branch of the federal government may review study materials.

QUESTIONS OR PROBLEMS: You will be told about any new information which might affect your decision about being in this research study. You will be given a copy of this consent form for your own records. You will always have the right to ask questions about this study at any time. If you have any questions about this study, please contact:

Dr. David Young - 420-6460 OR Dr. B. Anthony Armson - 420-6778

You can also contact the IWK Grace Research Services Office at 428-8765 for information about this study from an outside source.

I have read and understand this informed consent. I agree to participate in this research study. I realize that my participation is voluntary and that there is no guarantee that I will benefit from my involvement. I have been given the opportunity to ask questions and all my questions have been answered. I acknowledge that a copy of this consent form has been given to me.

________________	________________	________________
Signature of Participant	Printed Name of Participant	Date of Signature
Signature of Person Obtaining Consent	Printed Name of Person Obtaining Consent	Date of Signature

F:\research\consent.ind

Randomized Control Trial of Women with Indication for Induction of Labor

Patient presents for induction of labor
Determine eligibility for Misoprostol Study

Inclusion criteria:
- ≥ 37 wks gestation
- cephalic presentation
- live single fetus

Exclusion criteria:
- non-reassuring fetal heart rate tracing
- prior uterine surgery
- documented hypersensitivity to Misoprostol or other prostaglandins
- contraindication to vaginal delivery
- uncontrolled asthma or epilepsy

Eligible?

no → Do not enroll patient

yes → Explain study to patient
Invite patient to participate
Obtain consent - signed form

Select next envelope from box according to membrane status
- intact or ruptured

Oral Misoprostol

give 50 ug PO Q4H prn
until one of the following...
- progressive labor
- contraction frequency of 3 in 10 min
- NRFHR tracing
- delivery

Vaginal Misoprostol

give 25 ug PV
then 25 to 50 ug PV Q6H prn
until one of the following...
- progressive labor
- contraction frequency of 3 in 10 min
- NRFHR tracing
- delivery

Usual Induction Method

dinoprostone gel PV
and Oxytocin IV
as recommended by the attending physician

Physician or qualified nurse will reassess the patient before each administration of prostaglandin

All decisions regarding AROM, analgesia, epidural use and Oxytocin augmentation will be made by the attending physician.

For questions please contact Dr. David Young, pager 1342

Misoprostol Labour Induction Study

Principal Investigators:	Dr. DC Young	420-6460
	Dr. BA Armson	420-6778
Research Coordinator:	Cora Fanning	420-3158

Labour induction is a frequent obstetric intervention (~25%). Prostaglandins (PGs) are effective induction agents, but gastrointestinal (GI) intolerance has limited their use to intracervical and vaginal administration of dinoprostone (PGE_2) gels. These gels require refrigeration until use, and cost $40-50 per dose.

Misoprostol, a PGE_1 analogue, is marketed for oral treatment of upper GI disorders. The past decade has seen growing research on its use for induction of labour. Randomized controlled trials (RCTs) have compared both vaginal and oral administration of misoprostol to usual induction approaches. Misoprostol has been effective, well tolerated, and safe for mother/baby, though there is some evidence of increased frequency of excessive uterine activity. Cost per patient has been less than one-hundredth of other PGs, even less than intravenous (IV) oxytocin drip.

This project will advance research for term labour induction with a **three-group RCT** comparing **oral misoprostol** versus **vaginal misoprostol** versus **usual induction methods** here at the IWK Grace Health Centre. This trial will introduce misoprostol for induction of labour at this centre while providing new information by modifying interval of administration and broadening indications for use (ie. intravaginal misoprostol with ruptured amniotic membranes). Along with prior and ongoing research, this study could well serve as a pilot for a large RCT looking at more substantive outcomes (cesareans or neonatal morbidity) which will require a multicentre approach (sample size greater than 10,000 subjects), but which can be accomplished in Canada with appropriate external funding. Such a trial may well be needed before misoprostol can be recommended for non-research use.

Pregnant women scheduled for induction of labour will be asked to allow random assignment to one of three approaches. One group will receive oral misoprostol 50 μgs (half a 100-μg tablet), repeated at four-hour intervals until one of the following occurs: progressive labour, contraction frequency of 3 per 10 minutes, non-reassuring fetal heart rate (FHR) tracing, or delivery. The second group will receive vaginal misoprostol 25 μg initial dose, followed by 25 to 50 μg every six hours as needed for the same effect. The final group will be induced by the usual method here at the IWK Grace Health Centre (intravaginal or intracervical dinoprostone gel and IV oxytocin drip as recommended by the attending physician). All decisions with regard to artificial rupture of membranes, analgesia, epidural use, and oxytocin augmentation will be made by the attending physician.

The primary outcome is the time from randomization and induction to vaginal birth. Other outcomes address harm to the newborn (eg. cord blood acid base analysis and ACOG birth asphyxia criteria) and mother (Cesarean section, peripartum interventions), maternal GI intolerance and excessive uterine activity.

Subjects will have been seen in consultation by an obstetrician. Information pamphlets will be supplied to all physicians providing antepartum and intrapartum care at the IWK Grace Health Centre. Prenatal education providers will also receive this information. The informed consent process will be completed in the Perinatal Centre, the Fetal Assessment Unit, or on the arrival of the patient to the Labour/Delivery/Recovery Unit.

Patients are eligible if they have an indication for induction, a single fetus, in cephalic presentation and are at least 37 weeks gestation. Exclusion criteria will be: non-reassuring fetal heart rate tracing, prior uterine surgery, documented hypersensitivity to misoprostol or other PGs, or contraindication to vaginal birth.

Anticipated start date: April 6, 1999. Prior to this date we intend to provide your office with information suitable for sharing with potential participants. If we inadvertently miss you, please let the research coordinator know at the above number.

RESEARCH SUMMARY

MISOPROSTOL LABOUR INDUCTION STUDY

1. **Why is there a need to undertake this research?**

Labour induction is a frequent obstetric intervention (20-30%). Prostaglandins (PGs) are effective agents which are frequently used, but gastrointestinal (GI) intolerance has limited their use to intracervical and vaginal administration of dinoprostone (PGE_2) gels. These gels require refrigeration until use, and cost $40-50 per dose.

Misoprostol, a PGE_1 analogue, is marketed for oral treatment of upper GI disorders. The past decade has seen mushrooming literature on its use for induction of labour. Randomized controlled trials (RCTs) have compared both vaginal and oral administration of misoprostol to usual induction approaches. The principal investigator has led a research team at Memorial University of Newfoundland (MUN) which is one of two or three groups worldwide which has published RCTs on both oral and vaginal misoprostol for labour induction. Misoprostol has been found to be effective, well tolerated, and safe for mother/baby, though there is some evidence of increased frequency of excessive uterine activity. Cost per patient has been less than one-hundredth of other PGs, even less than intravenous (IV) oxytocin drip.

This project will advance our research for term labour induction with a three-group RCT comparing oral misoprostol versus vaginal misoprostol versus usual induction methods here at the IWK Grace Health Centre. This trial will introduce misoprostol for induction of labour at this centre while providing new information by modifying interval of administration and broadening indications for use (ie. intravaginal misoprostol with ruptured amniotic membranes). Along with prior and ongoing research, this study could well serve as a pilot for a large RCT looking at more substantive outcomes (cesareans or neonatal asphyxia) which will require a multicentre approach (sample size greater than 10,000 subjects), but which can be accomplished in Canada with appropriate external funding. Feedback received from a prior unsuccessful Medical Research Council of Canada Grant application in 1997 encouraged resubmission with these outcomes. Such a trial will be needed before misoprostol can be recommended for non-research use.

2. **What will the participant be asked to do?**

This is an RCT of pregnant women with an indication for induction of labour. Patients will be asked to allow random assignment to one of three approaches to initiation of labour induction. Study group allocation will be stratified based on membrane status (ruptured/intact). Sequentially numbered opaque envelopes will contain group assignment prepared using computer generated random number tables, in blocks of 4 and 6. One study group will receive by mouth misoprostol 50 μgs (half a 100-μg tablet), repeated at four-hour intervals until one of the following occurs: progressive labour, contraction frequency of 3 per 10 minutes, nonreassuring fetal heart rate (FHR) tracing, or delivery. The second study

34 lines

group will receive vaginal misoprostol 25 μg initial dose, followed by 25 to 50 μg every six hours as needed for the same effect. The final group will have induction of labour managed by the usual method here at the IWK Grace Health Centre (intravaginal or intracervical dinoprostone gel and IV oxytocin drip as recommended by the attending physician). All decisions with regard to artificial rupture of membranes, analgesia, epidural use, and oxytocin augmentation will be made by the attending physician. A staff physician will reassess the patient before each administration of prostaglandin.

The primary outcome will be the time from randomization and induction to vaginal birth. Other outcomes will include labour intervals to vaginal birth (time to full dilation, duration of membrane rupture, and labour stages), labour intervals to cesarean birth, frequency of maternal interventions and morbidity, birth route - vaginal (spontaneous, vacuum, or forceps) and cesarean, frequency of increased uterine activity (tachysystole or hyperstimulation), maternal satisfaction with labour evaluated by a questionnaire (Labour Agentry Scale), neonatal morbidity (cord artery acid base analysis, Apgar scores, ACOG criteria for birth asphyxia), and maternal GI intolerance (nausea, vomiting, diarrhea) by caregiver report and patient questionnaire.

3. **The usual accepted management:**

The accepted management at IWK Grace Health Centre for induction of labour involves a prostaglandin (dinoprostone gel) placed in the cervix or vagina, or an intravenous oxytocin drip (or both).

4. **The potential harms or benefits to the participants -**

The side effects of misoprostol are similar to those of dinoprostone and of similar low frequency from previous studies. These are largely gastrointestinal and include: nausea, vomiting, and diarrhea.

An important complication of any labour induction is increased uterine activity with possible adverse fetal effects. With oxytocin drips, these can usually be corrected within 10 minutes by stopping the oxytocin infusion. Use of prostaglandins, either intravaginally or orally, is more problematic. Attempts are made to remove the tablet remnants or gel from the vagina but have varying success. Removing an orally-administered tablet could be attempted by induced emesis. Neither of these approaches has proven very effective, hence labour and delivery units have a medical protocol to address the situation (intravenous ritodrine, a medication used in the treatment of preterm labour). Our experience to date has been that far less than 1:100 patients would need this treatment. Expeditious intervention for birth remains the final, though hopefully safely avoided, option.

The potential benefits for the patient include a more satisfying birth experience with less need of an IV drip, a more efficient labour, and fewer vaginal examinations.

5. **Who are the subjects and how will they be recruited?**

More than one thousand labour inductions take place each year at the IWK Grace. The most frequent indications for induction are: post-term pregnancy (› 41 weeks gestation), premature

34

rupture of membranes (PROM), maternal hypertension or pre-eclampsia, reduced amniotic fluid, intrauterine growth restriction (IUGR), and maternal diabetes mellitus. Subjects will be recruited from patients who are judged to have an indication for labour induction. All subjects will have been seen in consultation by a member of the Department of Obstetrics and Gynaecology. An information pamphlet and consent forms will be circulated to all family physicians providing antepartum and intrapartum care at the IWK Grace Health Centre, as well as to all obstetrician/gynaecologists. Prenatal education providers will be provided similar information. The informed consent process will be completed by an information session in the Perinatal Centre, the Fetal Assessment Unit, or on the arrival of the patient to the Labour/Delivery/Recovery Unit provided by the study nurse coordinator or staff physician (obstetrics/gynaecology consultant or resident).

6. **What are the inclusion and exclusion criteria?**

Patients will be eligible if they present with an indication for induction of labour, a single fetus, in cephalic presentation and at least 37 weeks gestation. Exclusion criteria will be: non-reassuring fetal heart rate tracing, prior uterine surgery, documented hypersensitivity to misoprostol or other prostaglandins, or contraindication to vaginal birth.

7. **Who are the researchers?**

The research team is made up of specialists from the Division of Maternal/Fetal Medicine, Department of Obstetrics and Gynaecology, IWK Grace Health Centre. None of this group has any financial interest in the company manufacturing misoprostol. There is no external funding being sought for this study. The principal investigator has considerable experience in RCTs of induction of labour and in particular with misoprostol. It is hoped that a refined protocol will be the basis of a national multicentred trial appropriate for external funding. To date, Searle Canada, the pharmaceutic manufacturer, has declined support of any research of misoprostol in the field of obstetrics; presumably because of medicolegal risks in obstetrics, controversy regarding pregnancy termination, and small market share!

8. **Safety monitoring -**

Detailed maternal and neonatal clinical and lab information will be collected on all study participants. A study investigator or nurse coordinator will be available by beeper at all times. Any serious adverse effect that occurs at our centre will be reported to the IWK Grace Research Office.

9. **How will consent be obtained?**

The great majority of patients who are judged to need induction of labour will be eligible. Consultation with an obstetrics and gynaecology specialist is a policy for induction in our centre with these indications. The attending physician will first approach the patient about the study, following determination of an induction indication, and confirm her suitability and willingness for participation. A patient who meets eligibility criteria for study enrollment will be interviewed to provide information concerning the study, the medication, and to have an opportunity to ask any questions. An information document and consent form will be provided for them to keep. This process may be carried out by a study investigator team member, a research nurse coordinator, or staff obstetrician/gynaecologist consultant, or resident. All this interaction will occur in the Health Centre. *

10. **Other issues -**

Discussion has occurred with the director of pharmacy who approves of this study. The study will not affect nursing personnel. There will be no effect on laboratory since laboratory investigation required for this study is part of normal lab studies for any patient with an indication of induction of labour (ie. cord artery acid base analysis). The data collection and monitoring of specific adverse events will be done by study research personnel.

(6)

CATEGORY **B**
GRANT APPLICATION

Applicant(s): Surname, given names	Department	Telephone Number of Principal Investigator
Principal Investigator: YOUNG, DAVID C.	OBSTETRICS/ GYNECOLOGY	420-6460
Co-Investigator(s): ARMSON, B. ANTHONY	Role in study Collaboration, supervision when PI away	

Budget Request: $15,000

Title: MISOPROSTOL LABOR INDUCTION STUDY

Outline of Research In Lay Terms: (12 lines or less)

Labor induction is a frequent obstetric intervention (≈20%). Prostaglandins (PGs) are effective agents, but gastrointestinal (GI) intolerance has limited use to non-oral routes. The traditional oxytocin "drip" requires intravenous (IV) use and discourages mobility. Misoprostol, a PG analogue, is marketed for oral treatment of GI disorders, but initiates uterine contractions, an undesirable GI side effect. Recently, there has been a research "boom" on vaginal misoprostol use in pregnancy to induce term labor drawing on this "side effect". The principal investigator has led one of three groups worldwide which has published on oral misoprostol to study effectiveness, GI tolerance, and safety for mother/ baby in term labor induction. Cost per patient has been less than one hundredth that of other PGs, even less than IV oxytocin. This project will advance our research for term labor induction with a three-group randomized controlled trial comparing oral to vaginal misoprostol and our center's established approach. We will assess time to vaginal birth, mother/baby well-being, mother's satisfaction, and GI effects. This trial may be a pilot for the externally-funded multicentre study needed before misoprostol can be approved for general use.

Signatures: Principal Investigator	Head of Department
	Name: DAVID YOUNG
Date: May 15, 1998	Date: May 15, 1998

One page summary:

Introduction

Labor induction is a frequent obstetric intervention (20→30%). Prostaglandins (PGs) are effective agents, but gastrointestinal (GI) intolerance has limited their use to intracervical and vaginal administration of PGE_2 gels.

Misoprostol, PGE_1 analogue, is marketed for oral treatment of upper GI disorders. The past five years has seen mushrooming literature on its use to initiate uterine contractions for pregnancy termination in the first and second trimesters, and labor induction in the third. Vaginal administration has been used almost exclusively, has been cost-effective (less than one hundredth that of PGE_2) and without demonstrated harm to mother or newborn. We have published a randomized controlled trial (RCT) on vaginal use. We have also published a 275-subject RCT of oral misoprostol versus a traditional induction regime (physician chosen combinations of intracervical or vaginal dinoprostone, intravenous (IV) oxytocin, and artificial membrane rupture). Oral misoprostol was effective, well tolerated, and without harm to mother or newborn. We have in press a double blind RCT of oral versus vaginal misoprostol in 206 subjects. Oral misoprostol was effective, though time to vaginal birth was 226 mins. longer, due to more time before labor was initiated. Oral misoprostol was associated with less uterine hyperstimulation ($P < 0.04$). We have also just completed an RCT of oral misoprostol versus IV oxytocin with term prelabor membrane rupture. Again, effectiveness was shown. There is no larger published collective experience with oral misoprostol labor induction.

Before embarking on a costly and necessarily multicenter RCT to evaluate more substantive outcomes (Caesareans or neonatal asphyxia) with sample size greater than 10,000, we seek funding for a three-group RCT of labor induction at term: oral misoprostol, vaginal misoprostol, and our center's established approach. Two other RCTs using differing administration schedules are about to begin by our group in St. John's.

Primary Research Question

and the cervix is unfavorable

When induction of labor at term is indicated, is there more than a four-hour difference in time to vaginal birth between vaginal misoprostol (25 μg initial dose, followed by 25 to 50 μg every six hours as needed), oral misoprostol (50 μg every four hours as needed), and the IWK Grace established protocol?

Secondary outcomes address harm to the newborn (including cord blood acid base analysis, and ACOG birth asphyxia criteria) and mother (Caesareans, peripartum interventions), maternal GI intolerance, and excessive uterine activity.

Research Plan

Eligible subjects will be at gestations greater than 37 completed weeks, with a cephalic presenting live single fetus, who have an indication for induction, and no contraindication to induction, vaginal birth, or PG use. Random allocation will be blocked and stratified (on membrane status).

Sample size calculations were based on $\Delta = 240$ minutes, α (2 tailed) = 0.05, $\beta = 0.05$, with σ from our prior publications. Adjustment for anticipated Caesareans (<20%) were made. Sample size is 510. Recruitment within a year is supported by our prior research (more than 1000 inductions per year at IWK Grace).

Name of Principal applicant and date of application: David C. Young May 15, 1998

Details of grant proposal: [Maximum 4 additional pages; 6(b)-6(e)] Use 12 pitch or larger, leave 3/4" margins.

For Operating grants:
- Introduction (Objectives, Hypothesis)
- Methods for study
- Procedures of analysis
- Anticipated outcome and significance
- Key reference

For Equipment request:
- Users of equipment
- Briefly describe research projects, hypothesis, goals will be used
- Non research use and application
- Describe any renovation or installation required

Title: **MISOPROSTOL LABOUR INDUCTION STUDY**

Introduction:

Labor induction is a frequent obstetric intervention (20→30%). Prostaglandin (PGs) are effective agents, but gastrointestinal (GI) intolerance has limited their use to intracervical and vaginal administration of PGE_2 gels.[1]

Misoprostol (Cytotec; Searle Canada, Oakville, Ontario, Canada) is an inexpensive synthetic PGE_1 analogue marketed in North America in an oral tablet form, which is stable at room temperature. Two formulations, 100 µg and 200 µg, are available on physician prescription for prevention and treatment of nonsteroidal anti-inflammatory drug induced gastric ulcers and treatment of duodenal ulcers.[2] The frequency of GI side effects is low with oral administration of up to 1600 µg per day. Misoprostol costs less than one hundredth the price per dose of commercial PGs currently used in labor induction.

Pharmacokinetic data for misoprostol use in pregnancy have now been published.[3] Plasma concentration peaks more quickly and to higher levels with oral ingestion, then fall rapidly. With vaginal administration, plasma level was sustained for ~~up to~~ beyond four hours. The authors suggest vaginal administration could (or should) be dosed at longer intervals than oral.

Vaginal Misoprostol for Labor Induction

Several RCTs have now shown vaginal placement of misoprostol tablets to be an effective method of inducing labor, without an increase in adverse maternal or neonatal outcomes.[4-14] In an RCT involving 222 subjects at term with membranes intact, our group[9] found decreased time to vaginal delivery (by approximately three hours), less frequent oxytocin augmentation, a strong trend for less use of epidural analgesia, and no difference in Caesarean births with misoprostol (50 µg every four hours). Neonatal outcomes, including cord blood acid base analysis, were not different. Median PG cost per patient with misoprostol was one hundredth that in the control subjects, who received our established induction protocol (physician-chosen combinations of intracervical or vaginal dinoprostone every six hours, artificial rupture of membranes (AROM), and oxytocin infusion).

Vaginal misoprostol was no less effective or safe than control groups in an RCT[12] of prelabor rupture of membranes (PROM) subjects. Two RCTs have been reported comparing different dose amounts (25 to 50 µg) and intervals (3 to 6 hours) of vaginal misoprostol.[13,14] Higher overall dosage is associated with more rapidly progressive labor, but higher rates of uterine hyperstimulation.

Oral Misoprostol for Labor Induction

An orally administered labor induction agent would likely be attractive to both patients and health care providers. Avoidance of intravenous lines in some parturients, and less frequent need for vaginal examination might be anticipated, and considered more client friendly. Greater freedom for upright positioning and ambulation might even facilitate labor progress.

Because misoprostol is known to be well tolerated orally when used for its primary indication, the management of upper gastrointestinal dysfunction, to study the effectiveness, safety and side effects of misoprostol as an oral agent for induction of labor seemed appropriate. We carried out an RCT[15] of oral misoprostol (50 μg every four hours if needed) versus our standard approach to term labor induction. The 275 women were randomized into strata based on membrane status. Prelabor rupture of membranes (PROM) had occurred in 56. In summary, the time from induction to vaginal birth with oral misoprostol was not significantly different from that with our established protocols. There were no clinically or statistically significant differences in maternal secondary outcome measures (Caesarean rate, frequency of epidural use, perineal trauma, or manual removal of the placenta). Neonatal outcomes, including cord blood acid base analysis, were not different. There was no difference in frequency of maternal GI side effects in the two groups.

Ngai et al[16] have recently reported a double blind RCT with a single 200 μg oral misoprostol dose versus placebo for cervical priming, in 80 women with PROM at term. The authors concluded that a single 200 μg misoprostol oral dose was effective for cervical priming, and may be effective for labor induction.[16] The oral misoprostol protocol and its purpose in our study was different from that of the Hong Kong investigators. Our median cumulative dose per subject for labor is similar to their single dose. It is reassuring that they did not find a significant problem with excessive uterine activity.

We now have in press a double-blind RCT[17] of oral versus vaginal misoprostol (50 μg every four hours as needed) in 206 term labor inductions. There was a shorter interval (846 versus 1072 minutes) to vaginal birth with vaginal application ($P = 0.004$), though no difference in active labor. The more frequent occurrence hyperstimulation ($P < 0.04$) in the vaginal group suggested optimal dosing interval for vaginal use remains to be determined. There was no difference in cesarean rate or neonatal outcomes.

An RCT from Egypt[18] of 40 near term inductions has reported similar findings to ours. They used a much higher dose of 100 μg every three hours and permitted a doubling of this dose. Our experience would caution against such an aggressive approach. They provided no neonatal outcome information.

The median PG cost in our previous studies[9,15] was $0.33 for misoprostol and a conservative $70.00 for standard therapy. Maximum PG expenses for any single patient were under $1.00 and more than $180.00 for misoprostol and standard therapy, respectively.

Fetal Safety Issues

Inherent in labor induction is the potential for excessive uterine activity which may interfere with uteroplacental perfusion sufficiently to compromise the fetus. Nonreassuring fetal heart rate changes associated with excessive uterine activity warrant intervention either to reduce uterine activity, provide more direct assessment of fetal well being (fetal blood sampling, other biophysical assessment), or removing the fetus from this environment (delivery), if simple measures such as position change or maternal oxygen supplementation are not corrective. When oxytocin induction results in uterine hyperstimulation with nonreassuring FHR changes, discontinuing the infusion can reduce oxytocin blood levels quickly. Use of vaginal dinoprostone gels or misoprostol is more problematic. Authors[19] describe vaginal lavage and removing tablet remnants, however, intravenous β- adrenergic agonist regimens have been used with apparent beneficial tocolytic response. Intravenous tocolysis is the approach necessary with oral misoprostol. Forced emesis would likely be ineffective, and distasteful. Our experience has been that far fewer than 1:100 patients would need IV tocolysis.

A recent overview[20] concludes misoprostol shows promise as a highly effective, inexpensive and convenient agent for labor induction, however, it cannot yet be recommended for routine use. Though no differences in perinatal outcome were found (in over 1700 subjects receiving vaginal misoprostol in RCTs), the increase in excessive uterine activity with fetal heart rate changes warrants the search for a more appropriate dose amount and/or interval.

No meta analysis has yet been published on use of oral misoprostol for labor induction. The evidence of effectiveness described above in the few existing RCTs is very encouraging. The incidence of hyperstimulation has been lower than that seen with either vaginal misoprostol or dinoprostone. Our double-blind RCT of oral versus vaginal misoprostol found lower rates with oral use.[17] This masked assessment provides the best possibility of unbiased review.

Despite these concerns of possible uterine hyperstimulation, substantive worrisome newborn outcomes (neonatal acidosis, meconium aspiration, and ACOG Birth Asphyxia criteria)[21] have been rare and not different between misoprostol and control groups. It is imperative that such outcome data be collected in future studies to quantitate more precisely potential risks of misoprostol (and traditional approaches).

Methods of Study:

Primary Research Question

When induction of labor at term is indicated, is there more than a four-hour difference in time to vaginal birth between vaginal misoprostol (25 μg initial dose, followed by 25 to 50 μg every six hours as needed), oral misoprostol (50 μg every four hours as needed), and the IWK Grace established protocol?

Secondary outcomes address harm to the newborn (including cord blood acid base analysis, and ACOG birth asphyxia criteria) and mother (Caesareans, peripartum interventions), maternal GI intolerance, and excessive uterine activity.

Research Plan

Patients will be eligible if they present with an indication for induction of labour, a single fetus, in cephalic presentation and at least 37 weeks gestation. Exclusion criteria will be: non-reassuring fetal heart rate tracing, prior uterine surgery, documented hypersensitivity to misoprostol or other prostaglandins, or contraindication to vaginal birth.

The most frequent indications for induction are: post-term pregnancy (› 41 weeks gestation), premature rupture of membranes (PROM), maternal hypertension or pre-eclampsia, reduced amniotic fluid, intrauterine growth restriction (IUGR), and maternal diabetes mellitus. Subjects will be recruited from patients who are judged to have an indication for labour induction. All subjects will have been seen in consultation by a member of the Department of Obstetrics and Gynaecology. An information pamphlet and consent forms will be circulated to all family physicians providing antepartum and intrapartum care at the IWK Grace Health Centre, as well as to all obstetrician/gynaecologists. Prenatal education providers will be provided similar information. The informed consent process will be completed by an information session on the arrival of the patient to the labour/delivery unit provided by the study nurse coordinator or staff physician (obstetrics/gynaecology consultant or resident).

Patients will be asked to allow random assignment to one of three approaches to initiation of labour induction. Study group allocation will be stratified based on membrane status (ruptured/intact). Sequentially numbered opaque envelopes will contain group assignment prepared using computer generated random number tables, in blocks of 4 and 6. One study group will receive misoprostol 50 μgs (half a 100-μg tablet), repeated at four-hour intervals until one of the following occurs: progressive labour, contraction frequency of 3 per 10 minutes, nonreassuring fetal heart rate (FHR) tracing, or delivery. The second study group will receive misoprostol 25 μg

initial dose, then 25 to 50 μg) placed in the vagina at six-hour intervals to the same effect. The final group will have induction of labour managed by the usual method here at the IWK Grace Health Centre (intravaginal or intracervical prostaglandin gel and IV oxytocin drip as recommended by the attending physician). All decisions with regard to artificial rupture of membranes, analgesia, epidural use, and oxytocin augmentation will be made by the attending physician. A staff physician will reassess the patient before each administration of misoprostol.

The primary outcome will be the time from randomization and induction to vaginal birth. Other outcomes will include labour intervals to vaginal birth (time to full dilation, duration of membrane rupture, and labour stages), labour intervals to cesarean birth, frequency of maternal interventions and morbidity, birth route - vaginal (spontaneous, vacuum, or forceps) and cesarean, frequency of increased uterine activity (tachysystole or hyperstimulation), maternal satisfaction with labour evaluated by a questionnaire (Labour Agentry Scale), neonatal morbidity (cord artery acid base analysis, Apgar scores, ACOG criteria for birth asphyxia), and maternal GI intolerance (nausea, vomiting, diarrhea) by caregiver report and patient questionnaire.

The proposed sample size[22] is 510 (3N). This is based on a clinically important difference $\Delta = 240$ min (from a patient survey before a prior study), ∝ (2 tailed) = 0.05, $\beta = 0.05$, with σ from our prior publications (588,524) and adjusting for anticipated cesareans (< 20%). The high power was chosen to increase our confidence in a no difference result.

Data Analysis:

Hypothesis testing will be performed only on the primary outcome. Other comparisons will be considered hypothesis generation. Presetting decision levels and hypotheses to be tested should minimize data dredging and post hoc significance bias. Analysis will be on an "intent to treat" basis. The primary outcome measure, time to vaginal birth, will be significant if $P<0.05$ (as per sample size calculation) using parametric statistics (Student's t).

ANOVA

Caesarean birth could not be included in the primary outcome analysis. Rank order nonparametric statistics using the Mann Whitney U test, where a Caesarean is a failure to deliver vaginally and ranked longer than any vaginal birth (as if infinite), would allow inclusion of all births in a secondary analysis with median time to vaginal birth as the measure of central tendency.

Kruskal Wallis

Secondary outcomes will be analysed by parametric and nonparametric (chi-square, Fisher exact, Mann Whitney U) statistics as appropriate. Continuous variables will be examined for normal distribution (Wilk-Shapiro/Rankit Plot) prior to using parametric statistics. Descriptive statistics will be used to assess demographic baseline data to confirm group comparability. Baseline data from nonvolunteers will be analysed to assess generalizability. The significance level for all secondary and hypothesis generating analyses will be 0.001 to account for multiple testing.

Anticipated Outcome and Significance:

With over 1,000 inductions per year at IWK Grace, this study is quite feasible in one year. Median PG doses per induction have been 2 to 3. With 340 patients receiving misoprostol rather than dinoprostone (~ $50 per dose), there would be a pharmacy saving of more than $30,000 while the study is ongoing.

The results of this study, other studies with different dosing now in progress in St. John's, and our past published work should form the basis of an MRC proposal for a multicentre RCT on a more substantive outcome, such as cesarean rate (N = 3500 to detect a change from 12% to 9%) or neonatal asphyxia (1% to 2%). A previous proposal to MRC suggested high chance of support for such study with a substantive outcome.

References:

1. Keirse MJNC, Chalmers I. Methods of inducing labour. In: Chalmers I, Enkin M, Keirse MJNC, eds. Effective care in pregnancy and childbirth. Oxford University Press, 1989; 1057-79.
2. Garris RE,Kirkwood CF, Misoprostol: A prostaglandin E_1 analogue. Clinical Pharmacy 1989; 8:627-44.
3. Zieman M, Fong SK, Benowitz NL, Banskter D, Darney PD. Absorption kinetics of misoprostol with oral or vaginal administration. Obstet Gynecol 1997; 90:88-92.
4. Sanchez-Ramos L, Kaunitz AM, Del Valle GO, Delke I, Schroeder PA, Briones DK. Labor induction with the prostaglandin E_1 methyl analogue misoprostol versus oxytocin: A randomized trial. Obstet Gynecol 1993; 81:332-6.
5. Fletcher HM, Mitchell S, Simeon D, Frederick J, Brown D. Intravaginal misoprostol as a cervical ripening agent. Br J Obstet Gynaecol 1993; 100:641-4.
6. Fletcher HM, Mitchell S, Frederick J, Simeon D, Brown D. Intravaginal misoprostol versus dinoprostone as cervical ripening and labour-inducing agents. Obstet Gynecol 1994; 83:244-7.
7. Wing DA, Jones MM, Rahall A, Goodwin TM, Paul RH. A comparison of misoprostol and prostaglandin E_2 gel for preinduction cervical ripening and labor induction. Am J Obstet Gynecol 1995; 172:1804-10.
8. Wing DA, Rahall A, Jones MM, Goodwin TM, Paul RH. Misoprostol: An effective agent for cervical ripening and labor induction. Am J Obstet Gynecol 1995;172:1811-6.
9. Mundle WR, Young DC. Vaginal misoprostol for induction of labor: randomized controlled trial. Obstet Gynecol 1996; 88:521-5.
10. Wing DA, Ortiz-Omphroy G, Paul RH. A comparison of intermittent vaginal administration of misoprostol with continuous dinoprostone for cervical ripening and labor induction. Am J Obstet Gynecol 1997; 177:612-8.
11. Sanchez-Ramos L, Peterson DE, Delke I, Gaudier FL, Kaunitz AM. Labor induction with prostaglandin E_1 misoprostol compared with dinoprostone vaginal insert: a randomzied trial. Obstet Gynecol 1998; 91:401-5.
12. Sanchez-Ramos L, Chen AH, Kaunitz AM, Gaudier FL, Delke I. Labor induction with intravaginal misoprostol in term premature rupture of membranes: a randomized study. Obstet Gynecol 1997; 89:909-12.
13. Wing DA, Paul RH. A comparison of differing dosing regimens of vaginally administered misoprostol for pre-induction cervical ripening and labor induction. Am J Obstet Gynecol 1996; 175:158-64.
14. Farah LA, Sanchez-Ramos L, Rosa C, Del Valle GO, Gaudier FL, Delke I, Kaunitz AM. Randomized trial of two doses of prostaglandin E_1 analogue misoprostol for labor induction. Am J Obstet Gynecol 1997; 177:364-71.
15. Windrim R, Bennett K, Mundle W, Young DC. Oral administration of misoprostol for labor induction: a randomized controlled trial. Obstet Gynecol 1997; 89:392-7.
16. Ngai SW, To WK, Lao T, Ho PK. Cervical priming with oral misoprostol in pre-labor rupture of membranes at term. Obstet Gynecol 1996; 87: 923-6.
17. Bennett KA, Butt K, Crane JMG, Hutchens D, Young DC. A masked randomized comparison of oral and vaginal administration of misoprostol for labor induction. Obstet Gynecol 1998 (in press).
18. Toppozada MK, Anwar MYM, Hassan HA, El-Gazaerly. Oral or vaginal misoprostol for induction of labor. Int J Gynecol Obstet 1997; 56:135-9.
19. Egarter CH, Husselein PW, Rayburn WF. Uterine hyperstimulation after low dose prostaglandin E2 therapy: tocolytic treatment in 181 cases. Am J Obstet Gynecol 1990; 163:794-6.
20. Hofmeyr GJ. Misoprostol administered vaginally for cervical ripening and labour induction with a viable fetus. In: Neilson JP, Crowther CA, Hodnett ED, Hofmeyr GJ (eds.) Pregnancy and Childbirth Module of the Cochrane Database of Systematic Reviews (updated 2 Dec. 1997), The Cochrane Collaboration; Issue 1. Oxford: Update Software; 1998.
21. ACOG Committee Opinion. Fetal distress and birth asphyxia. 137 April 1994. The American College of Obstetricians and Gynecologists, Washington, DC.
22. Meinert CL. Sample Size, Number of Treatment Groups. In: Clinical Trials - Design, Conduct, and Analysis. Oxford University Press, 1986, p. 74.

Misoprostol Labor Induction Study

Demographics

Study # __________ Name __________ __________________ Hosp # __________ Group _____

Age _____ G___ P___ A___ Induction Date ____________ (YYYY/MM/DD) Time ______ (HRS)

Height _____ (Cm) Weight _____ (Kg) Gr B Strep. status: pos neg

Indication for induction:
- >41 wks ____
- PROM ____
- IUGR ____
- mat. diabetes ____
- ↑BP ____
- preeclampsia ____

Bishop's Score

Factor	**0**	**1**	**2**	**3**	
Dilation (cm)	0	1-2	3-4	5-6	____
Effacement (%)	0-30	40-50	60-70	80	____
Station	-3	-2	-1 or 0	+1 or +2	____
Consistency	Firm	Med	Soft	__	____
Position	Post	Mid	Ant	__	____
			**Total Score**		

Interventions

IV used: Y N

Epidural used: Y N
Narcotic type ______________ # doses _____

PV exams ____

Prostaglandin used:
Misoprostol ____
Dinoprostone ____

	Dose 1	*Dose 2*	*Dose 3*	*Dose 4*	*Dose 5*
route	______	______	______	______	______
dose	______	______	______	______	______

Total # doses ______
Total dose used ______

Oxytocin used: Y N # min ______

Maternal GI upset: nausea ____ vomiting ____ diarrhea ____

Delivery

Time:		*elapsed time from induction (min)*
onset of induction	______	
ROM	______	______
onset 1st stage	______	______
onset 2nd stage (fully dilated)	______	______
end 2nd stage (delivery)	______	______
end 3rd stage	______	______

Type of delivery:
- SVD ____
- Vacuum ____
- Forceps ____
- C/S ____
  - due to.... NRFHR____
  - FTP ____
  - Other __________

Episiotomy: nil ____ m lat ____ mid ____

Laceration: none ____ perineal ____ vaginal ____ cervical ____ sphincter ____ mucosa ____

Manual removal placenta: Y N

Blood loss: normal (≤500) ____ increased (>500) ____ decreased ____

Maternal morbidity: Tachysystole _____ Hyperstimulation _____ Anemia _____ Blood Tx ____ Fever ____ Other ___________

Baby

Gender: M F weight ________ (gms) Apgar (1min) ____ (5min) ____ meconium: Y N

scalp pH ______
cord pH ______
cord BE ______

Neonatal morbidity: ACOG criteria for birth asphyxia _____
(Apgar 5min ≤3, cord pH ≤7.00, base deficit ≥16)
____
____

BIRTH RECORD

Grav ☐ Para ☐
Ab ☐ SB ☐ NND ☐
EDC ☐ Gest ☐
Preg/Med Complications:

Membrane Rupture:
❑ SRM Date: ☐
❑ Suspected Time: ☐
❑ ARM Duration: ☐
Meconium: ❑ No ❑ Yes
Time first noted: ☐
❑ Maternal fever >38 in labour

Initiation/Progress of Labour:
❑ Spontaneous Onset
❑ Oxytocin Augmentation
❑ Induction Reason: ☐

Cervical Ripening/Induction:
❑ Prostaglandin
and/or
❑ Oxytocin

1ST STAGE ESTABLISHED: Date: ☐ Time: ☐

2ND STAGE ONSET: Date: ☐ Time: ☐

BIRTH: Date: ☐ Time: ☐ Position at Birth: ☐
❑ Spontaneous ❑ C/S Reason: ☐
❑ Forceps (&/or) ❑ Vacuum
❑ Mid ❑ Mid
❑ Low ❑ Low
❑ Outlet ❑ Outlet
❑ Attempted Only ❑ Attempted Only

❑ Rotation
❑ Manual
❑ Forceps

❑ Other Intervention (e.g. Breech Extraction): ☐

PLACENTAL DELIVERY: Date: ☐ Time: ☐
❑ Spontaneous ❑ Assisted ❑ Manual
Umbilical Vessels: ❑ 3 ❑ 2 Cord pH Done: ❑ No ❑ Yes
Abnormalities Describe: ☐ Weight: ☐
Oxytocic: ❑ No ❑ Yes Type: ☐ Dose: ☐ Route: ☐
Infusion postpartum: ☐
PPH (>500ml) ❑ No ❑ Yes Estimated blood loss: ☐ *ml*

Episiotomy:
❑ None
❑ Midline
❑ Mediolateral

Lacerations:
❑ None
❑ 1st° (vaginal)
❑ 2nd° (perineal)
❑ 3rd° (anal sphincter)
❑ 4th° (rectal mucosa)
Suture required: ❑ No ❑ Yes
Count Verified: ❑ Sutures ❑ Sponges

Analgesia/Anaesthesia:
❑ None ❑ Narcotic ❑ Spinal
❑ Nitrous Oxide ❑ Epidural ❑ General
❑ Other: ☐

Medications (to mother within 24 hrs. before birth)

Time	Drug / Dose / Route

BABY: ❑ Girl ❑ Boy Weight ☐ (g)

**APGAR**	0	1	2	1 min.	5 min.	10 min.
**Heart Rate**	Absent	Below 100	Above 100			
**Resp. Effort**	Absent	Slow Irreg.	Good Crying			
**Muscle Tone**	Limp	Some Flexion	Active Motion			
**Reflex Irritab.**	None	Grimace	Cough Sneeze			
**Colour**	Blue Pale	Body Pink Blue Extre.	All Pink			
**APGAR SCORE TOTALS**						

Erythromycin Eye Ointment: ____________
Signature

Resuscitation:
❑ None ❑ Oxygen only
Pos. Pressure: ❑ With Mask ❑ With ET Tube
❑ Chest Compressions
Tracheal Suctioning: ❑ No ❑ Yes
Meconium below Cords: ❑ No ❑ Yes
Duration of Resuscitation: ☐
Medications used: ☐

	<10 sec.	10-60 sec.	>1 min.
Age at First Breath:	❑	❑	❑
Age at First Cry:	❑	❑	❑
Age at Sustained Resp.	❑	❑	❑

Signature of Nurse/Phys. responsible for resuscitation

❑ Stillbirth Date/Time last FHR: ☐
Date/Time last FM: ☐

Comments:

RCP/04 - 04/98

Signature of Nurse Attending Birth

Signature of Physician Attending Birth

White - Mother's Chart / Yellow - Newborn's Chart / Pink - Physician's Office

BIRTH RECORD

Uterine Hyperstimulation
Protocol for Medical Management

Definitions:

Hyperstimulation:

- late fetal heart rate decelerations, fetal tachycardia ≥160 bpm or any other non reassuring fetal heart rate (NRFHR) tracing in association with hypertonic contraction or tachysystole

  Hypertonic contraction:
  - contraction with a duration of ≥90 seconds

  Tachysystole:
  - more than 5 contractions greater than 50mm Hg in a ten minute period

Immediate actions:

- Stop IV oxytocin infusion
- Administer O_2 at 5-10 l/m by mask
- Place patient in left lateral position
- Pelvic examination
  - Remove agent if possible (tablet remnants or gel)
- Establish IV access (if not already in place)

Medical management:

- If no improvement after above actions carried out, and no contraindication exists:
  - **administer Ritodrine 6mg in 10ml normal saline IV PUSH over 2-3 minutes**
- If non reassuring fetal heart rate (NRFHR) persists, prepare for possible C-section and notify the anaesthetist of tocolytic given

Repetitive hypertonic contractions or tachysystole would preclude
- a further dose of dinoprostone or misoprostol
- an increase in oxytocin

but

would not warrant medical management as outlined above

[illegible]

MISOPROSTOL LABOUR INDUCTION STUDY

QUESTIONNAIRE

STUDY NUMBER ______________

HOW TO FILL OUT THIS QUESTIONNAIRE

In responding to each question, please circle a number from 1 to 7. The circled number should indicate to what extent you are in agreement or in disagreement with the statement.

Here is an example of how to respond to this questionnaire.

EXAMPLE: **I FELT CONFIDENT**

The circled number corresponds to what you felt during the birth experience. If you felt that you were **"OFTEN"** confident during your labour, circle the number 2. If you were **"RARELY"** confident during the birth experience, circle the number 7. Circle just one number for each question.

	Almost always						Rarely
1. I felt confident: **Almost always**	(1)	2	3	4	5	6	7
2. I felt confident: **Often**	1	(2)	3	4	5	6	7
3. I felt confident: **A little more than half the time**	1	2	(3)	4	5	6	7
4. I felt confident: **About half the time**	1	2	3	(4)	5	6	7
5. I felt confident: **Slightly less than half the time**	1	2	3	4	(5)	6	7
6. I felt confident: **Sometimes**	1	2	3	4	5	(6)	7
7. I felt confident: **Rarely**	1	2	3	4	5	6	(7)

NOW TRY TO REMEMBER WHAT YOU FELT DURING YOUR RECENT BIRTH EXPERIENCE

1. I experienced complete awareness of everything that was happening.

1 2 3 4 5 6 7
Almost Always ... Rarely

2. I felt fearful.

1 2 3 4 5 6 7
Almost Always ... Rarely

3. I did not know what to expect from one moment to the next.

1 2 3 4 5 6 7
Almost Always ... Rarely

4. Everything seemed wrong.

1 2 3 4 5 6 7
Almost Always ... Rarely

5. I felt powerless.

1 2 3 4 5 6 7
Almost Always ... Rarely

6. I experienced great anxiety.

1 2 3 4 5 6 7
Almost Always ... Rarely

7. I had a feeling of constriction and of being confined.

1 2 3 4 5 6 7
Almost Always ... Rarely

8. I had a sense of being in control.

1 2 3 4 5 6 7
Almost Always ... Rarely

9. I experienced a sense of conflict.

1 2 3 4 5 6 7
Almost Always ... Rarely

10. I felt open and receptive.

1 2 3 4 5 6 7
Almost Always ... Rarely

11. I felt like I was falling to pieces.

1 2 3 4 5 6 7
Almost Always ... Rarely

12. I felt important.

1 2 3 4 5 6 7
Almost Always ... Rarely

13. I felt relaxed.

1 2 3 4 5 6 7
Almost Always ... Rarely

14. I felt secure.

1 2 3 4 5 6 7
Almost Always ... Rarely

15. I felt capable.

1 2 3 4 5 6 7
Almost Always ... Rarely

16. I felt tense.

1 2 3 4 5 6 7
Almost Always ... Rarely

17. I felt good about my behaviour during childbirth.

1 2 3 4 5 6 7
Almost Always ... Rarely

18. I experienced a sense of success.

1 2 3 4 5 6 7
Almost Always ... Rarely

In a similar way, please circle the most appropriate for you during this labour.

		Not At All							Almost Always
1.	I was nauseated	0	1	2	3	4	5	6	7
2.	I vomited	0	1	2	3	4	5	6	7
3.	I had diarrhea	0	1	2	3	4	5	6	7

If you needed a labour induction in another pregnancy, would you want to have the same induction method?

Yes No

If not, can you say why?
