# Supplementary material for: Oral misoprostol, low dose vaginal misoprostol, and vaginal dinoprostone for labor induction: Randomized controlled trial
Source: PLoS One. 2020 Jan 10;15(1):e0227245. doi: 10.1371/journal.pone.0227245 (PMC6953875; doi:10.1371/journal.pone.0227245)
Supplement: S1 Data Dictionary — (PDF) [file pone.0227245.s003.pdf]

**File Name C:\Users\Owner\Documents\ObGyn\Labor Induction\Misoprostol\Manuscript\Data Dictionary Miso RCT PLOS.sx**

| | |
|---|---|
| Variables | 111 |
| Selected Cases | 511 |
| Omitted Cases | 0 |
| Total Cases | 511 |

| **Variable** | **Data Type** | **Format** | **Variable Label/Value Labels** |
|---|---|---|---|
| MSTATUS | ANSI String 10 | A10 | Membrane status |
| MStaRG | Integer | A 6 | Membrane status regression<br>0 Ruptured 1 Intact |
| STUDYNO | ANSI String 10 | A10 | Study Number |
| TACY20TD | Integer | A 6 | Tachysystole 20 minutes TD<br>0 No 1 Yes |
| HYPERTD | Integer | A 6 | Hyperstimulation TD<br>0 No 1 Yes |
| GRP | ANSI String 10 | A10 | Study Group |
| Group | Integer | A 6 | Study Group<br>1 PO 2 PV 3 USUAL |
| AGEYRS | Integer | A 6 | maternal age (years) |
| GRAVIDA | Integer | A 6 | Gravity |
| PARA | Integer | A 6 | Parity |
| Parous | Integer | A 6 | Parous<br>0 No 1 Yes |
| GEST | Integer | A 6 | Gestational age (weeks) |
| WGT | Real | A 9 | Weight (kg) |
| HGT | Integer | A 6 | Height (cm) |
| GBS | ANSI String 10 | A10 | Group B Strep |
| ANTIBIO | ANSI String 10 | A10 | Recieved antibiotics |
| INDICATN | ANSI String 26 | A26 | Induction indication |
| DILATION | Integer | A 6 | Dilation |
| EFFACE | Integer | A 6 | Effacement |
| STATION | Integer | A 6 | Station |
| CONSIST | Integer | A 6 | Consistency |
| POSITION | Integer | A 6 | Position |
| BISHOPS | Integer | A 6 | Bishops Score |
| LOBISH | Integer | A 6 | Low Bishops Score<br>1 Yes 2 No |
| IVUSED | ANSI String 10 | A10 | IV used |
| EPIDURAL | ANSI String 10 | A10 | Epidural Received |
| PVEXAMS | Integer | A 6 | Number of pelvic exams |
| NARCOTIC | ANSI String 26 | A26 | Narcotics recieved |
| NARC_DOS | Integer | A 6 | Number of narcotic doses |
| MISDOSES | Integer | A 6 | Number of misoprostol doses |
| MISAMT | Integer | A 6 | Misoprostol amount (mcg) |
| DINDOSES | Integer | A 6 | Dinoprostone doses |
| DINAMT | Integer | A 6 | Dinoprostone amount (mg) |
| PGDOSES | Integer | A 6 | Prostaglandin doses |
| OXYUSED | ANSI String 10 | A10 | Oxytocin received |
| OXYDUR | Integer | A 6 | Oxytocin duration (minutes) |
| NAUSEA | ANSI String 10 | A10 | Nausea |
| VOMITING | ANSI String 10 | A10 | Vomited |
| DIARRHEA | ANSI String 10 | A10 | Diarrhea |
| ROMHOW | ANSI String 10 | A10 | How membranes ruptured |
| ONSETST1 | Real | A 9 | Time to onset stage one (minutes) |
| TIMTOFUL | Integer | A 6 | Time to full dilation (minutes) |
| STAGE1 | Integer | A 6 | Stage one duration (minutes) |
| STAGE2 | Integer | A 6 | Stage two duration (minutes) |
| DELIVERY | Integer | A 6 | Delivery by cesarean<br>1 No 2 Yes |
| TIMTODEL | Integer | A 6 | Time to Delivery (minutes) |
| TIMRANK | Real | A 9 | Time to Delivery (min) CS ranked longest |
| VAG12 | Integer | A 6 | Vaginal birth < 12 hours<br>1 yes 2 No |
| VAG24 | Integer | A 6 | Vaginal birth < 24 hours<br>1 yes 2 No |
| VAG48 | Integer | A 6 | Vaginal birth < 48 hours<br>1 yes 2 No |
| VAG72 | Integer | A 6 | Vaginal birth < 72 hours<br>1 yes 2 No |

| | | | |
|---|---|---|---|
| CeSect | Integer | A 6 | Delivery by CS (1 or 2) |
| | | | 1 Yes 2 No |
| CeSect2 | Integer | A 6 | Delivery by CS (0 or 1) |
| | | | 0 No 1 Yes |
| VagBir | Integer | A 6 | Vaginal Birth (0 or 1) |
| | | | 0 No 1 Yes |
| WHY_CS | ANSI String 19 | A19 | Why cesarean |
| EPISIOTM | ANSI String 10 | A10 | Episiotomy method |
| LACERATN | ANSI String 10 | A10 | Laceration type |
| INTACT | Integer | A 6 | Intact perineum (0 or 1) |
| | | | 0 No 1 Yes |
| MANPLACT | ANSI String 10 | A10 | Manual placenta removal |
| BLDLOSS | ANSI String 10 | A10 | Blood loss amount postpartum |
| MAT_MORB | ANSI String 26 | A26 | Maternal morbidity |
| GENDER | ANSI String 10 | A10 | Newborn sex |
| BABYWT | Integer | A 6 | Newborn weight (grams) |
| APGAR1 | Integer | A 6 | Apgar score at one minute |
| LOAP1 | Integer | A 6 | Low Apgar at one min (1 or 2) |
| | | | 1 <7 2 7 plus |
| APGAR5 | Integer | A 6 | Apgar score at five minutes |
| LOAP5 | Integer | A 6 | Low Apgar at five min (1 or 2) |
| | | | 1 <7 2 7 plus |
| LOAPGR | Integer | A 6 | Very low Apgar at 5 min (0 or 1) |
| | | | 0 4 plus 1 < 4 |
| MECONIUM | ANSI String 10 | A10 | Meconium |
| SCALPPH | Real | A 9 | Scalp pH value |
| CORDPH | Real | A 9 | Cord artery pH |
| PH715 | Integer | A 6 | Cord artery pH < 7.15 (0 or 1) |
| | | | 0 No 1 < 7.15 |
| LOPH | Integer | A 6 | Cord artery pH < 7.00 (0 or 1) |
| | | | 0 No 1 < 7.00 |
| CORDBE | Real | A 9 | Cord artery base excess |
| LOXS | Integer | A 6 | Cord artery base excess < -12 |
| | | | 0 No 1 < -12 |
| XLOXS | Integer | A 6 | Cord artery base excess < -16 |
| | | | 0 No 1 < -16 |
| BABYMORB | ANSI String 40 | A40 | Newborn morbidity |
| STUDY | ANSI String 10 | A10 | Study number |
| QES1 | Integer | A 6 | LAS(18) Question 1 |
| | | | 1 Almost always 7 Rarely |
| QES2 | Integer | A 6 | LAS(18) Question 2 |
| QES2A | Integer | A 6 | LAS(18) Question 2A |
| QES3 | Integer | A 6 | LAS(18) Question 3 |
| QES4 | Integer | A 6 | LAS(18) Question 4 |
| QES4A | Integer | A 6 | LAS(18) Question 4A |
| QES5 | Integer | A 6 | LAS(18) Question 5 |
| QES5A | Integer | A 6 | LAS(18) Question 5A |
| QES6 | Integer | A 6 | LAS(18) Question 6 |
| QES6A | Integer | A 6 | LAS(18) Question 6A |
| QES7 | Integer | A 6 | LAS(18) Question 7 |
| QES7A | Integer | A 6 | LAS(18) Question 7A |
| QES8 | Integer | A 6 | LAS(18) Question 8 |
| QES9 | Integer | A 6 | LAS(18) Question 9 |
| QES9A | Integer | A 6 | LAS(18) Question 9A |
| QES10 | Integer | A 6 | LAS(18) Question 10 |
| QES11 | Integer | A 6 | LAS(18) Question 11 |
| QES11A | Integer | A 6 | LAS(18) Question 11A |
| QES12 | Integer | A 6 | LAS(18) Question 12 |
| QES13 | Integer | A 6 | LAS(18) Question 13 |
| QES14 | Integer | A 6 | LAS(18) Question 14 |
| QES15 | Integer | A 6 | LAS(18) Question 15 |
| QES16 | Integer | A 6 | LAS(18) Question 16 |
| QES16A | Integer | A 6 | LAS(18) Question 16A |
| QES17 | Integer | A 6 | LAS(18) Question 17 |
| QES18 | Integer | A 6 | LAS(18) Question 18 |
| SATIS | Integer | A 6 | LAS(18) Score |
| NAUSEA_D | Integer | A 6 | I was nauseated ( 0 to 7) |
| | | | 0 Not at all 7 Almost always |
| VOMITED | Integer | A 6 | I vomited (0 to 7) |
| | | | 0 Not at all 7 Almost always |
| HAD_DIAR | Integer | A 6 | I had diarrhea (0 to 7) |
| | | | 0 Not at all 7 Almost always |
| DO_AGAIN | ANSI String 10 | A10 | I would want same induction method |
