# Supplementary figures and images for: Oral misoprostol, low dose vaginal misoprostol, and vaginal dinoprostone for labor induction: Randomized controlled trial
Source: PLoS One. 2020 Jan 10;15(1):e0227245. doi: 10.1371/journal.pone.0227245 (PMC6953875; doi:10.1371/journal.pone.0227245)

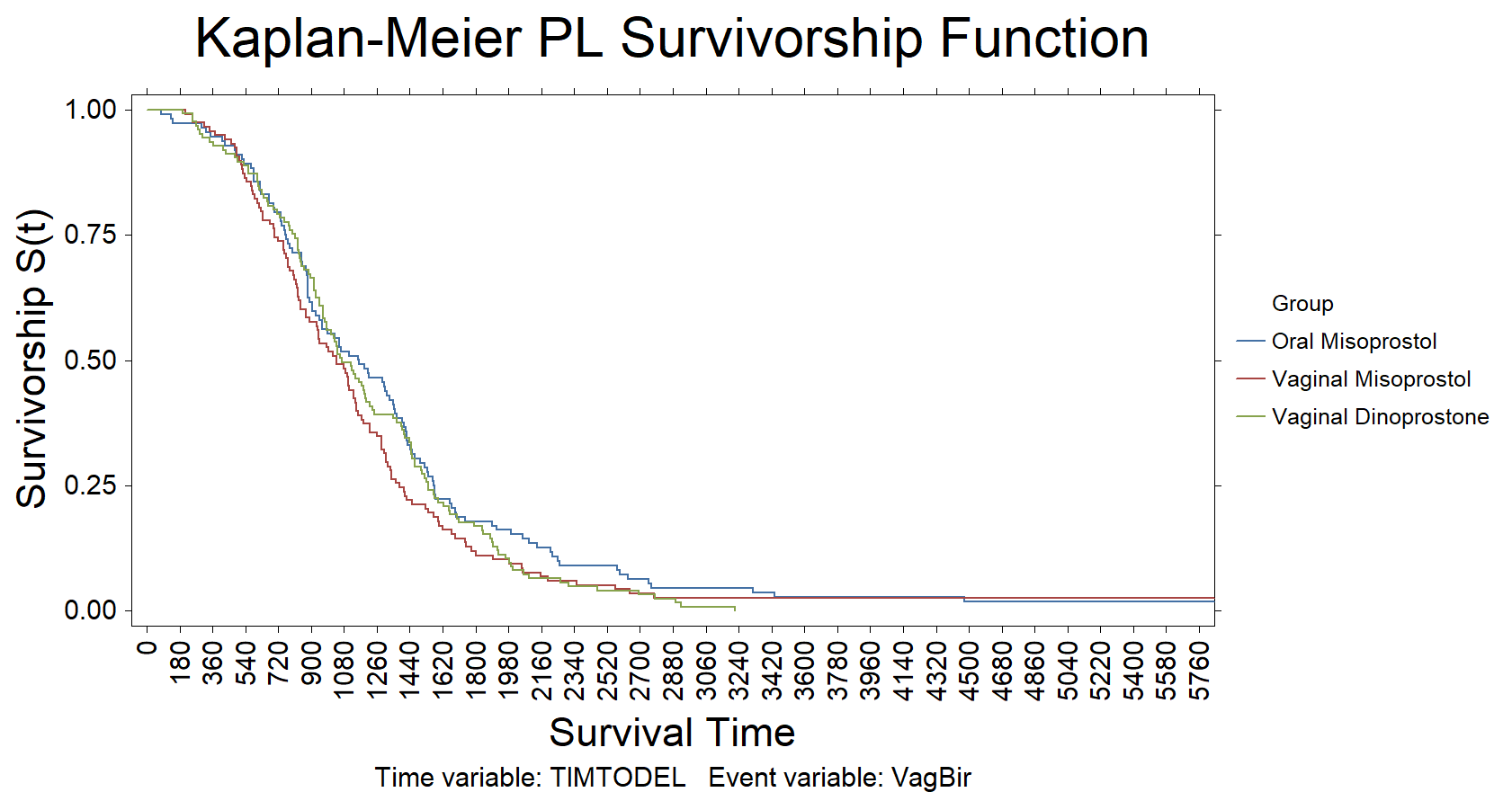

Supplement: S1 Fig — Y-axis: proportion of subjects not yet with vaginal birth. X-axis: time since induction begun (minutes). Time variable: TIMTODEL-time to vaginal birth. Cesarean births are censored. Event variable: VagBir-vaginal birth. Plot is truncated at 5760 minutes (4 days). Hence, for oral misoprostol, 2 vaginal births and 2 cesareans, and, for vaginal misorpostol, 3 vaginal births and 2 cesareans are not represented. (TIF) [file pone.0227245.s004.tif]

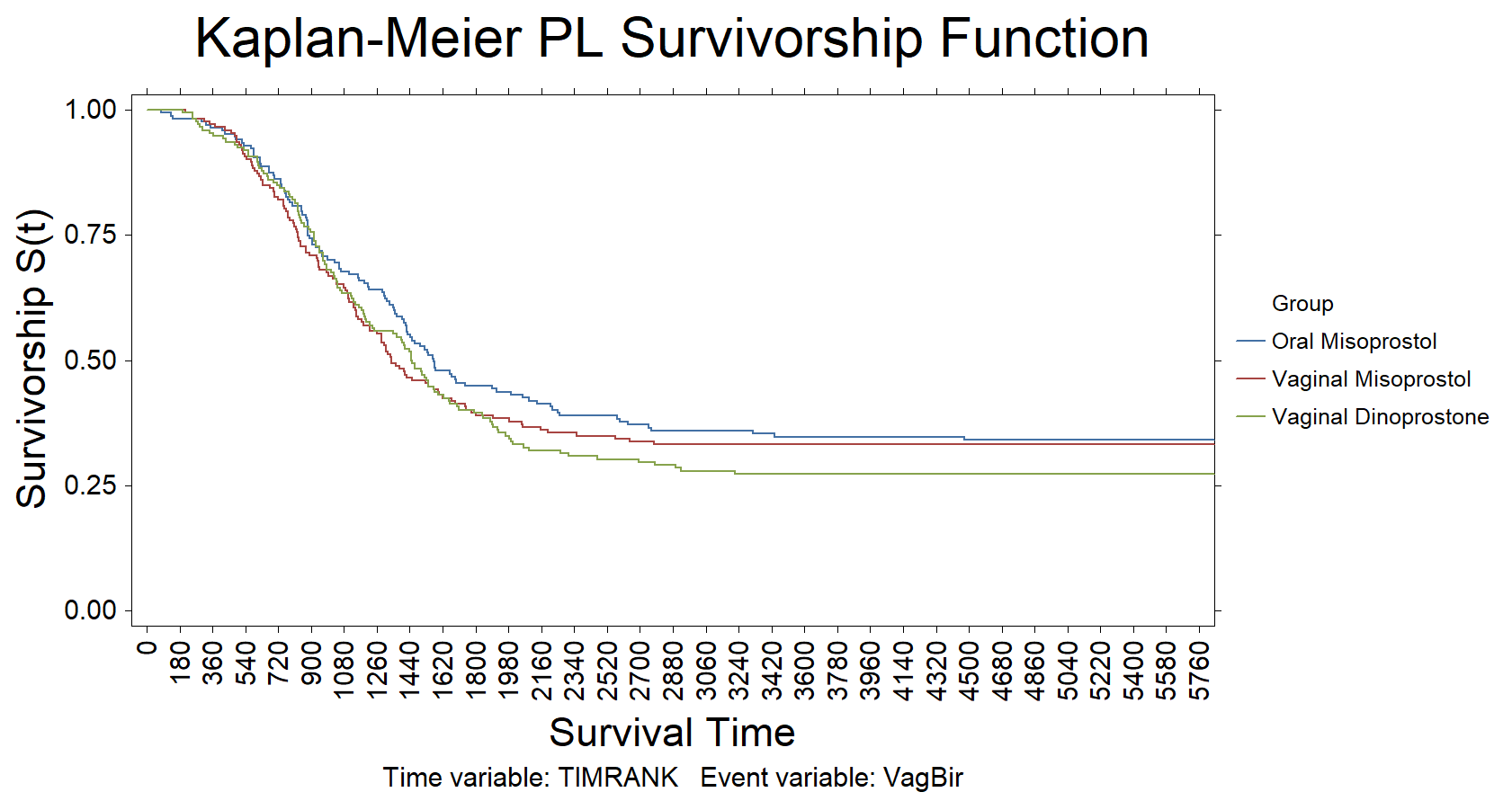

Supplement: S2 Fig — Y-axis: proportion of subjects not yet with vaginal birth. X-axis: time since induction begun (minutes). Time variable: TIMRANK-time to vaginal birth. Cesarean births ranked longest Event variable: VagBir-vaginal birth. Plot is truncated at 5760 minutes (4 days). Hence, for oral misoprostol, 2 vaginal births, and, for vaginal misoprostol, 3 vaginal births are not represented. (TIF) [file pone.0227245.s005.tif]
